# The exosome: a review of current therapeutic roles and capabilities in human reproduction

**DOI:** 10.1007/s13346-022-01225-3

**Published:** 2022-08-18

**Authors:** Marko Dimik, Pevindu Abeysinghe, Jayden Logan, Murray Mitchell

**Affiliations:** 1 Centre for Children’s Health Research, School of Biomedical Sciences, Faculty of Health, Queensland University of Technology, Level 8, 62 Graham Street, South Brisbane, QLD 4101 Australia; 2 Centre for Children’s Health Research, Leader – Child and Reproductive Health Group, Centre for Immunology and Infection Control, School of Biomedical Sciences, Faculty of Health, Queensland University of Technology, Brisbane, Australia

**Keywords:** Drug delivery, Exosomal biomarkers, Exosome cargo loading, Nanotherapeutics, Nanomedicine

## Abstract

Exosomes are nano-vesicles (30–150 nm) which may be useful as therapeutic delivery vehicles and as diagnostic biomarkers. Exosomes are produced naturally within the human body and therefore are not prone to immunogenicity effects which would otherwise destroy unelicited foreign bodies. Clinically, they have been regarded as ideal candidates for applications relating to biomarker developments for the early detection of different diseases. Furthermore, exosomes may be of interest as potential drug delivery vehicles, which may improve factors such as bioavailability of loaded molecular cargo, side effect profiles, off-target effects, and pharmacokinetics of drug molecules. In this review, the therapeutic potential of exosomes and their use as clinical biomarkers for early diagnostics will be explored, alongside exosomes as therapeutic delivery vehicles. This review will evaluate techniques for cargo loading, and the capacity of loaded exosomes to improve various reproductive disease states. It becomes important, therefore, to consider factors such as loading efficiency, loading methods, cell viability, exosomal sources, exosome isolation, and the potential therapeutic benefits of exosomes. Issues related to targeted drug delivery will also be discussed. Finally, the variety of therapeutic cargo and the application of appropriate loading methods is explored, in the context of establishing clinical utility.

**Graphical abstract:**

Exosomes have more recently been widely accpeted as potential tools for disease diagnostics and the targeted delivery of certain therapeutic molecules–and in due time exosomes will be utilised more commonly within the clinical setting. Specifically, exosomal biomarkers can be identified and related to various detrimental conditions which occur during pregnancy. Considering, this review will explore the potential future of exosomes as both diagnostic tools and therapeutic delivery vehicles to treat related conditions, including the challenges which exist towards incorporating exosomes within the clinical environment to benefit patients.

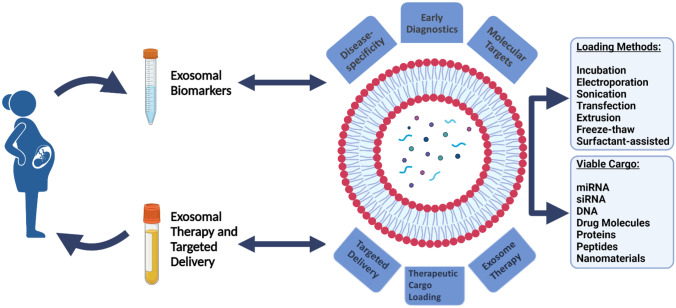

## Exosomes: an introduction to their origin, diversity, and cellular roles

Exosomes are classified as small (30–150 nm), phospholipid bilayer extracellular vehicles (EVs), which are released by both eukaryotic and prokaryotic cells for the purpose of intercellular communication and signalling [1, 2]. Initially, exosomes were identified as EVs used for excreting unwanted cellular waste–however, further research has found that exosomes are important molecular mediators in cellular communication through their ability to transport proteins, metabolites, and various nucleic acids across the body [3, 4]. In support of this, recent literature has identified specific binding proteins which contribute to cellular transportation machineries related to different exosomal RNA species and regulatory proteins interaction such as with syntenin and ADP-ribosylation factor 6 (ARF6) [3, 5]. Exosomes can be formed and secreted by cells through various stepwise processes relating to the initial formation of endosomes and the inward budding of multi-vesicular bodies [3–5].

Exosomes are secreted by many different cell and tissue types, including macrophages, placental tissue, epithelial cells, endometrial cells, uterine cells, follicular fluid, embryos, oviductal epithelium, dendritic cells, cancer cells, and mesenchymal stem cells [5, 6]. They are also abundantly found in various bodily fluids such as breast milk, amniotic fluid, plasma, saliva, semen, and cervical-vaginal fluid–signalling their applicability as biomarkers through portraying the physiological state of different donor cells [3, 6]. For example, exosomal RNA differs from that of the parent cell and recently various shared and selective RNA binding proteins have been identified and implicated in processes relating to RNP complexes which are involved in cellular regulation relating to RNA translation, transport, maturation, and metabolism [3].

Due to their functions of transporting cellular materials and reflecting the physiological state of the donor cell, exosomes can influence various processes relating to inflammation, central nervous system (CNS) communication, immune responses, and different types of tissue repair [2]. Their phospholipid bilayer membrane contains lipid structures such as ceramides and cholesterol, which assists in the sorting, secretion, and signalling between the exosome and host cell [7]. Exosomes also contain mRNA, DNA, non-coding RNA, ribosomal RNA, transfer RNA, and small nuclear RNA. The abundance of RNA species within exosomes plays a pivotal role in various biological processes, particularly in different disease states and disease progression factors [8–10]. Hence, exosomes may be considered important therapeutic tools for the possible treatment and/or early detection of various diseases, including reproductive and pre-natal disorders [7–10].

## Exosomal formation and maturation

**Fig. 1:**
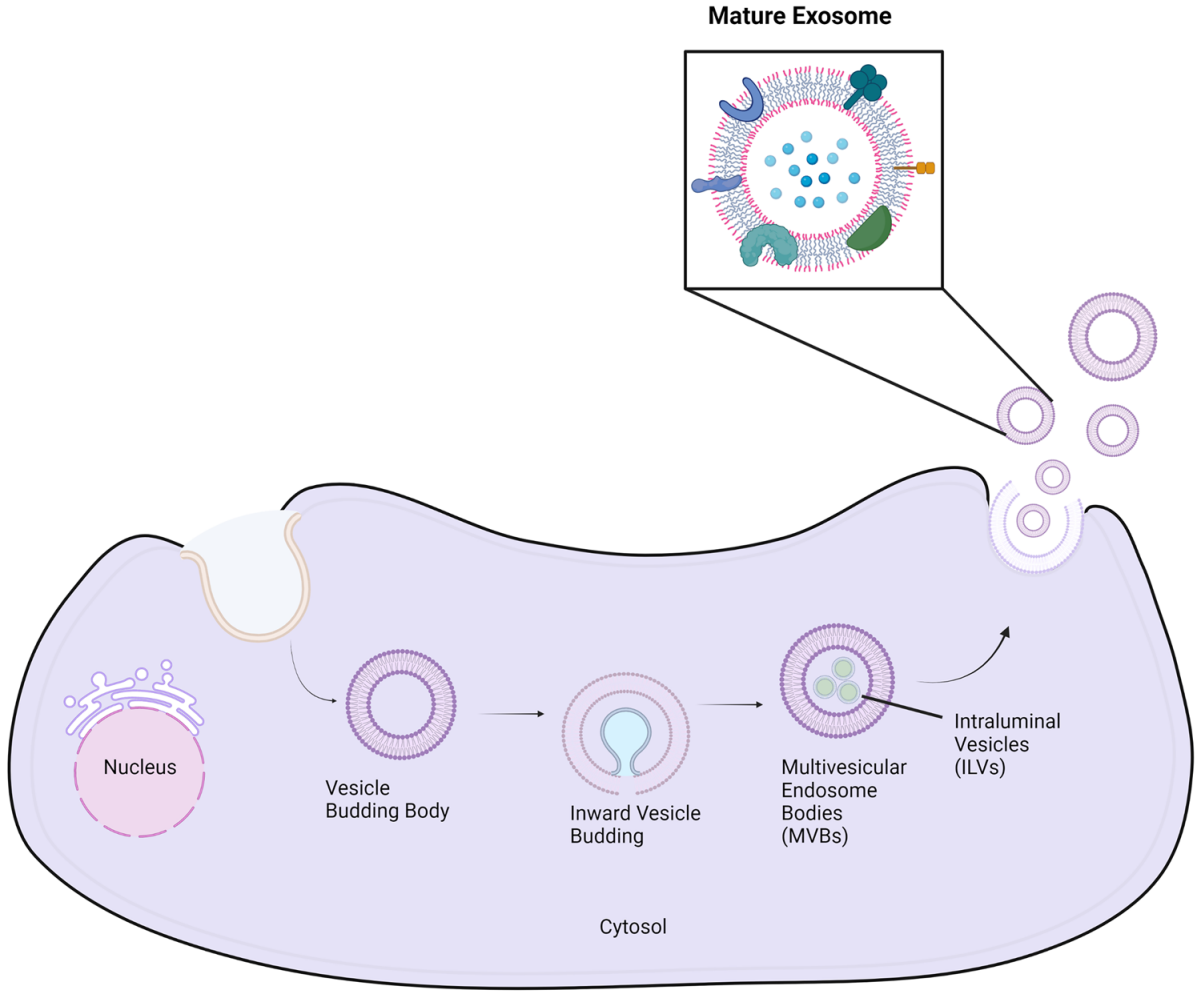
The proposed process of exosome formation, including the production of the MVB followed by the ILV, then further followed by endocytosis and the eventual inward budding of the MVB membrane releasing mature exosomes into the extracellular environment

Exosomes are formed from late endosomes, which are created by the inward budding of the multivesicular body membrane (MVB) (see Fig. 1)–two main processes have been suggested regarding the formation of exosomes [11]: (1) the ESCRT pathway and (2) endocytosis and recycling of cargo back into MVBs. The budding of the late-stage endosomal membrane creates clusters of intraluminal vesicles (ILVs) within the MVBs. Throughout the process, various proteins are added within the developing membrane while different cytosolic components are engulfed and encapsulated inside the ILVs [12]. The whole developmental stage is mediated and promoted through the endosomal sorting complex required for transport (ESCRT) to facilitate MVB production, vesicle budding, and the cargo sorting of various constituents [12, 13]. Recently, it has been found that ILV formation is highly dependent on ESCRT-related constituents such as Rab11, Vps4a, and ATPase [13]. Many of the ILVs are eventually released into the extracellular space following fusion with the plasma membrane–eventually becoming characteristic of a typical spherical exosome [14].

This sorting complex consists of four main proteins, being ESCRT-0 to ESCRT-3, and this mechanism is activated by the recognition and segregation of ubiquitinated proteins to various domains in the endosomal membrane through the ubiquitin-binding sites of the ESCRT-0 component [15]. Following, there are multiple interactions between the ESCRT-1 and EXCRT-2 complexes, which further promotes the combining with ESCRT-3–the protein component involved in the budding process [15]. The final step in this proposed mechanism includes the cleaving of the buds to form ILVs while ESCRT-3 disconnects from the MVB membrane via energy supplied by the Vps4 sorting protein [16].

The other prominent exosomal cargo sorting and biogenesis pathway suggested within literature is independent of the ESCRT system and is involved with the direct inward budding of the cell membrane [17]. The process relies on complex lipids such as ceramides which can self-associate into raft-like structures to then bind with the initial membrane structure and contribute towards the budding process–resulting in ILVs [12, 18]. These raft-like structures contain high concentrations of sphingomyelinases which assist in the production of ceramides through hydrolytically removing phosphocholine moieties on sphingomyelin [19]. Related literature has supported this mechanism by showing that a neutral sphingomyelinase inhibitor is an effective method to inhibit the secretion of exosomes [20]. Similarly, it has also been found that inhibition of ESCRT machinery can promote exosome release through lysosomal dysfunction, while the inhibition of ceramide suppressed basolateral CD9- and CD63-positive exosome release [12, 19]. As ceramide is formed through the removal of phosphocholine moieties by sphingomyelinases, these results may indicate that ceramide is highly involved in the formation of specific types of exosomes and their release [12, 19].

It has also been reported that tetraspanin proteins are involved in both exosomal cargo loading, sorting, and production [21], with tetraspanins such as CD9, CD81, and CD63 being some of the most prominent exosomal surface markers for detection [22]. It has also been found that certain lysosomal transmembrane proteins (LAMP1/2, PLD3) were found exclusively in CD63-predominant exosomes, while soluble lysosomal enzymes (CTSV/D/C, TPP1) were present in both CD63 and CD9 [22]. This could suggest a mechanism more structured towards membrane-specific sorting, involving one or multiple tetraspanins [22].

The tetraspanin-enriched microdomain (TM) is considered as the ubiquitous membrane platform and is important for the sorting of various receptors and signalling proteins within different compartments of the plasma membrane [22, 23]. The CD81 tetraspanin together with the TM are specifically involved in chaperoning important cellular communication-related receptors towards exosomes [24]. Additionally, the mechanisms which facilitate the chaperoning of different receptors and the various tetraspanins depend on the cellular origin of exosomal biosynthesis [25]. Furthermore, the protein referred to as “ALIX” has been found to be involved in the selective secretion of exosomes through collaborating with tetraspanins and thus controlling the sorting of specific proteins and downstream pathways [25, 26]. A notable difference between tetraspanin-dependent sorting and ALIX-dependent sorting is that tetraspanins require specific regulation through ubiquitination sites, while ALIX is both ubiquitin-dependent and ubiquitin-independent within its binding capacities [26, 27]. Table 1 shows the comparative differences and related literature regarding exosomal formation.

**Table 1:** The various genomic markers involved in exosomal formation and the related recent literature

**Protein name**	**Type of cells utilised in literature**	**Isolation method**	**Exosomal markers**	**Inhibitory constituents for confirmation**	**References**
**ESCRT-0**					
HRS	HeLa	Ultracentrifugation	CD63	shRNA	[151]
	HeLa CIITA	Tangential flow filtration, PEG-6000 precipitation, ultracentrifugation	CD63, LMP1, CD81	shRNA	[152]
	Nasopharyngeal carcinoma HK1	PEG-6000 precipitation, ultracentrifugation	Syntenin-1	shRNA	[152]
STAM1	HeLa CIITA	PEG-6000 precipitation, ultracentrifugation	CD63, MHCII	shRNA	[152]
STAM2	HEK-293	Differential centrifugation	VPS4, CD63, CD9	shRNA	[153]
**ESCRT-I**					
TSG101	HeLa CIITA	PEG-6000 precipitation, ultracentrifugation	CD63, MHCII	shRNA	[152]
	Caco-2	Differential centrifugation	Wnt5b	siRNA	[154]
	Neuroblastoma SH-SY5Y	Differential centrifugation, ultracentrifugation	Flot1, Tsg101	siRNA	[155]
VSP28	*Drosophila* S2	Ultracentrifugation	Wg, CD63	siRNA	[156]
**ESCRT-II**					
EAP20	Jurkat E4	Ultracentrifugation, differential centrifugation	CD63	siRNA	[157]
EAP45	U1	Ultracentrifugation, differential centrifugation	CD63	siRNA	[157]
**ESCRT-III**					
CHMP2A	MCF-7	Ultracentrifugation, differential centrifugation	SDC1CTF, syntenin, CD63	siRNA	[158]
CHMP4	MCF-7	Ultracentrifugation, differential centrifugation	SDC1CTF, syntenin, CD63	siRNA	[158]
**Associated proteins**					
ALIX	HeLa-CIITA	Tangential flow filtration, PEG-6000 precipitation, ultracentrifugation	CD63, CD81, LMP1	shRNA	[152]
	MCF-7	Ultracentrifugation, differential centrifugation	SDC1CTF, syntenin, CD63	siRNA	[158]
	HT29, HCT116	Ultracentrifugation	ALIX, Rab5, Rab7, Rab11	shRNA	[159]
VPS4	MCF-7	Ultracentrifugation, differential centrifugation	SDC1CTF, syntenin, CD63	Dominant negative VPS4 overexpression	[158]
	Huh7.5	Ultracentrifugation, ultrafiltration	CD63, Hsp70	siRNA	[160]
	MDCK	Differential centrifugation	ALIX, Tsg101, Hrs	Dominant negative VPS4 overexpression	[16]
**ESCRT-independent mechanisms**					
RAB31	HEK-293 T	Ultrafiltration	CD63, HRS, FLOT1, FLOT2	shRNA	[123]
Ceramides	Neuro 2a	Differential centrifugation	CD63, ALIX, flotillin-1	GW4869 ceramide inhibitor	[161]
	A549	Differential centrifugation	TSG101, CD81, CD61, CD9	GW4869 ceramide inhibitor	[162]
	RAW264.7	Differential centrifugation	CD61, CD81	GW4869 ceramide inhibitor	[163]
Flotillin-2	PC-3	Ultracentrifugation, differential centrifugation	TSG101, CD81, CD61, CD9, ALIX	siRNA	[164]
	Human Astrocytes	Density gradient ultracentrifugation	Flotillin, HSP90	Aβ1–42	[165]
PLD2	MCF-7	Differential centrifugation	Syntenin, CD63	siRNA	[5]
ARF6	MCF-7	Differential centrifugation	Syntenin, CD63	siRNA	[5]

### Exosomal functions, cellular interactions, and clinical applicability

Exosomes have been found to be increasingly involved in cellular messaging and complex cell-to-cell communication [28]. Characteristically, exosomal communication (including paracrine, endocrine, exocrine, or synaptic-based) is primarily used by cells when more complex and distant communication must be established with other related cells [28]. Several recognition marker proteins are involved in the communicative processes, including the tetraspanin complexes and various Rab proteins (Rab27A/B) [28]. For example, exosomes released by a tumour or other cells can bind to nearby cells and travel through the bloodstream to another part of the body to deliver cellular information related to the biological state of the donor cell [29]. This is completed by the host cell absorbing the exosome through fusion with the membrane, receptor-mediated engulfment, and endocytosis mechanisms [29].

**Fig. 2:**
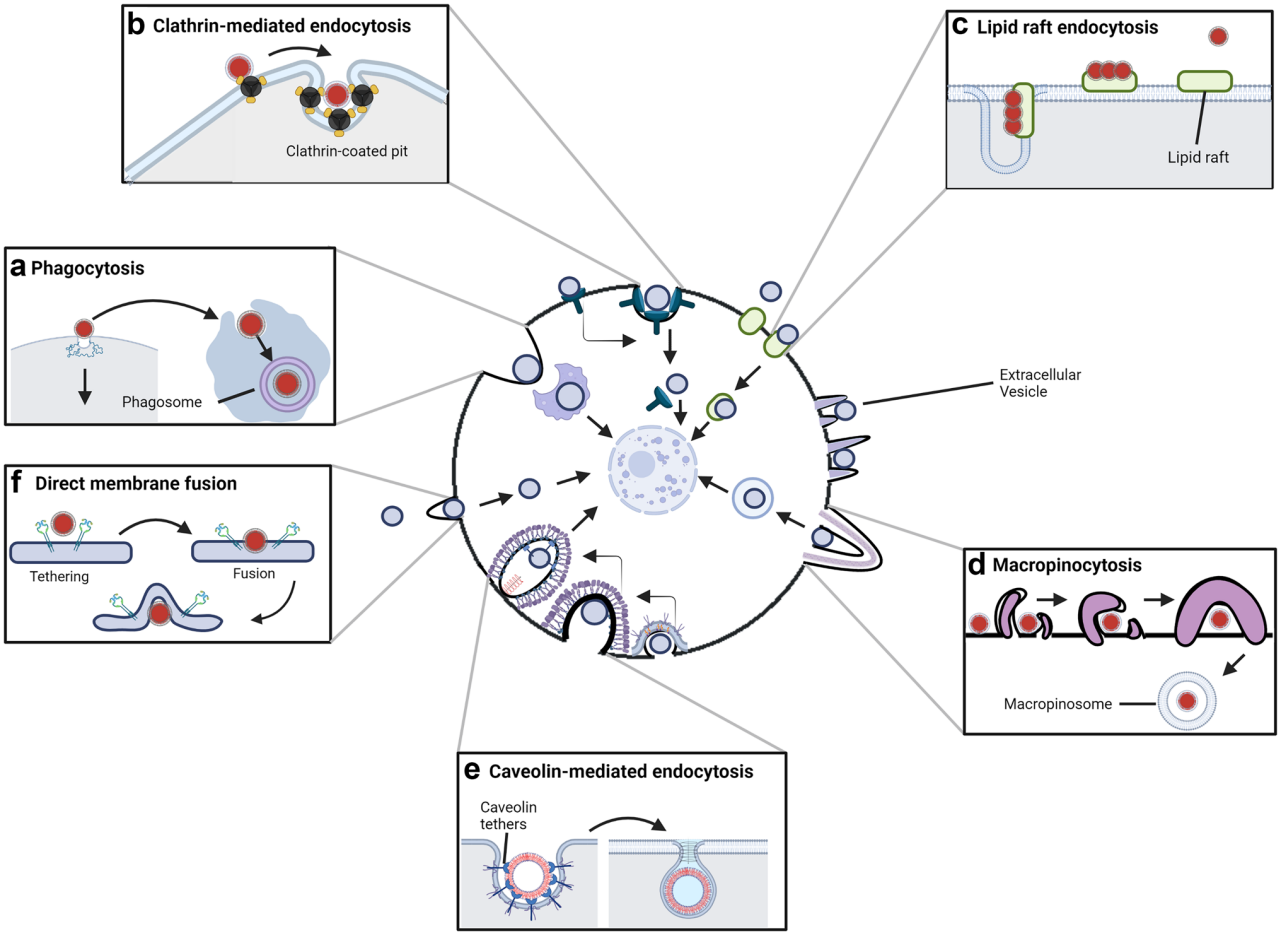
Exosomal cellular communication and the processes by which the exosome may be introduced into the host cell. **a** This shows the process of phagocytosis, wherein the EV is engulfed by the cell [30, 31]. **b** Clathrin-mediated endocytosis includes the process of internalising the EV through the assembly of clathrin-coated vesicles and lipid rafts [28]. **c** The process of lipid raft-mediated endocytosis, where the EV is chaperoned into the cell through binding with a lipid raft [30]. **d** Engulfing the EV via macropinocytosis, an endocytic pathway in which membrane ruffles form and fuse into the intracellular environment [31]. **e** Caveolin-mediated endocytosis in which small sub-domains of glycolipid raft indentations called caveolae are formed and can internalise EVs into the cell, acting as a type of chaperoning system [30, 31]. **f** Membrane fusion wherein the EV can directly fuse with the plasma membrane of the cell and become internalised rapidly [31]. Adapted from previous research [31]

It is evident to note that the biodistribution of exosomes is determined by a variety of factors, such as the nature of the donor cell, the targeted cellular destination, and the delivery pathway. Largely, the biodistribution of exosomes from their cellular origin to the targeted site has been described as asymmetric, wherein the physiological condition of the host may affect exosomal biodistribution throughout the body [30, 31]. Depending on the cellular environment, exosomes may interact with membrane proteins to initiate vital signalling pathways leading to internalisation. Other mechanisms include phagocytosis, micropinocytosis, and various types of endocytosis mediated through clathrin, caveolin, and lipid-raft mediums (see Fig. 2 for reference), meaning that exosomes may be taken up by the receiving cell through different methods and rates [31]. Hence, the consideration of factors such as exosomal biodistribution and pharmacokinetic parameters for clinical applicability is pivotal for assessing therapeutic parameters such as the half-life of circulating exosomes loaded with therapeutic cargo [31].

The presence of exosomal surface proteins such as integrins assists in guiding exosomes to their target cell, thereby facilitating a type of intrinsic cellular targeting [32, 33]. The multitude of cellular signalling mechanisms which exosomes contain allow for flexibility in cellular transport and recognition, which may support their function as potential biomarkers and therapeutic cargo vectors [32]. Additionally, exosomal cargo can influence both the internal and external environments of the recipient cells. Such environmental changes can include activating or deactivating signalling pathways through various phosphorylation events and transferring genetic material between different cells [32]. An example of this can be noted during pregnancy, where exosomal signalling can mediate inflammatory pathways, growth factors, foetal development, and activation of the maternal-foetal vascular exchange pathway [34].

Considering the multitude of functions exhibited by exosomes, recent literature has elicited considerable interest towards their clinical applicability relating to the transfer of various types of cargo between cells within the body. As exosomes exhibit enhanced immunocompatibility, a phospholipid bilayer structure protecting them from degradation, and flexibility in the types of contained cargo, they could be considered as potential diagnostic biomarkers and therapeutic vehicles for cargo such as proteins, compounds, and drug molecules [35]. Furthermore, their relatively small size and membrane structure allows them to travel through a multitude of areas within the human body, including the ability to safely pass through the blood–brain barrier (BBB) [8, 9].

The various methods for exosomal cargo loading is an important topic to consider when discussing the many different types of possible cargo and therapeutic targets. Previous research has led to the viable use of pre-clinical exosomal models and their potential uses. In example, the medication paclitaxel (PTX) has been loaded into exosomes multiple times and found to produce higher therapeutic activity with notably reduced side effects and increased bioavailability when utilising exosomes as delivery vehicles [36]. The potential of exosome-mediated cytotoxic cancer therapy therein resides in the ability for exosomes to safely deliver a molecule to the target site while minimising medication side effects and potential harm to healthy neighbouring cells [36]. This aspect can also relate to maximising bioavailability through protecting the cargo from first-pass metabolism and/or enzymatic degradation [36].

## Exosomal changes in reproductive disorders

As exosomes are highly involved in cell-to-cell communication and exosomal content is relative to the state of the donor cell, they may be utilised as potential biomarkers in dysfunctions and diseases during human reproduction [37]. In human reproduction, many processes are highly regulated and require specific steps and cellular support to undergo normal completion. Such can include follicular growth, oogenesis, implantation, embryonic development, fertilisation, and foetal development [37]. Exosomes are involved in the progression of the reproductive cycle relating to various regulatory mechanisms initiated through foetal-maternal communication and cellular regulation–all of which act to adapt the body to the many different physiological changes [37]. Such mechanisms can be related to immunological responses, inflammatory signals, and metabolic adaptations necessary for nurturing a growing foetus [37, 38]. The expression of quintessential mechanisms is mediated through various exosomal and cellular communication relating to direct cellular contact, hormonal signalling, and EV transportation throughout the maternal circulation [38].

In the healthy individual, these processes are tightly regulated in space and time to carry out the necessary alterations required for normal reproductive modifications to occur. Hence, the role of the exosome becomes important in maintaining a conserved reproductive state due to their functions in cellular communication and regulation through the different stages of the reproductive cycle [37, 38]. However, many of these normal processes can be dysregulated, leading to conditions such as localised or widespread inflammation, polycystic ovarian syndrome (PCOS), premature ovarian failure (POF), endometriosis, gestational diabetes mellitus (GDM), and pre-eclampsia [37].

Changes often occur at a cellular level before signs and symptoms of pregnancy complications begin to manifest [37]. Many such changes can be affected by gestational age, genetic factors, and individual health status–all important factors in the variations to exosomes and their cargo through the different stages of pregnancy [39]. Circulating exosomes contain complex mixtures of RNA species, proteins, and lipids, which are capable of modifying ‘normal’ biological functions and exerting downstream effects such as premature immune cell activation, inflammatory cytokine release, and dysregulation of pivotal circulating hormones [39].

Exosomal communication requires maternal exosomes to travel through the villous space in the placenta to regulate cargo and can be measured within the peripheral blood of pregnant women from the 6th week of gestation, particularly the exosomes which originate in the trophoblast area [40]. As exosomes carry cellular information from the donor cell around the peripheral circulation, the cellular cargo which is exclusively related to the donor cell is an important factor in distinguishing which biological functions may have been altered [40]. Such alternations can be related to the contents of exosomal cargo, especially that of miRNA and various proteins, and can often be related to a physiological change which may indicate a pathological state [41].

It can be common for complications during pregnancy to result in various physiological changes for both the mother and the foetus and increase the risk of congenital conditions and miscarriage [39]. In example, pre-eclampsia can result in an increased risk of cerebral palsy and early-onset hypertension and diabetes, while GDM may also increase the risk of early-onset metabolic dysfunction later in life [42–44]. As reproductive conditions contain many altercations on a cellular level, exosomal biomarkers can be important in distinguishing the occurrence and development of such pathological conditions during pregnancy [38, 39, 45].

## Exosomal functions in the healthy reproductive cycle

Exosomes carry through many different functions relating to the regulation of multiple physiological functions within pregnancy towards ensuring positive outcomes during the reproductive process [37, 39]. During the early stages of reproduction and the menstrual cycle, exosomes are abundantly found in the uterine luminal fluid [37]. As cell-to-cell signalling becomes even more crucial during pregnancy and reproduction, exosomes exhibit various specific roles regarding the regulation of pre-conceptive cellular mechanisms [37].

### Exosomes in the fertilisation process

During the process of fertilisation, the spermatozoa migrate from the vagina to the oviducts during the formation and development of a viable blastocyst for implantation into the endometrial surface of the uterus, located in the uterine cavity [37]. Previous research has found that uterine fluids are rich in cellular components such as proteins, RNA species, and lipids–much of which is contained in exosomes [37]. During the fertilisation and implantation processes, both physiological and morphological changes occur wherein the uterine and endometrial tissue and receptivity changes, along with cellular signalling and the involvement of various hormones and other stimuli [38, 39, 46]. Relatedly, the endometrium is involved in the early stages of implantation during the reproductive process and contains many proteins and fluids to assist this process [46]. For example, epithelial-mesenchymal transition and decidualisation is regulated through increasing levels of ovarian steroids and hormones such as oestrogen and progesterone and other proteins [47]. Relatedly, exosomes involved in the chaperoning of different proteins have been found to be taken in and released from the endometrium [46]. Exosomes derived from the trophoblast have been associated with modulating endometrial receptivity through various transcriptomic alterations, which ultimately may assist in both the pre- and post-fertilisation processes [46, 47].

### Exosomes in the placental development process

**Fig. 3:**
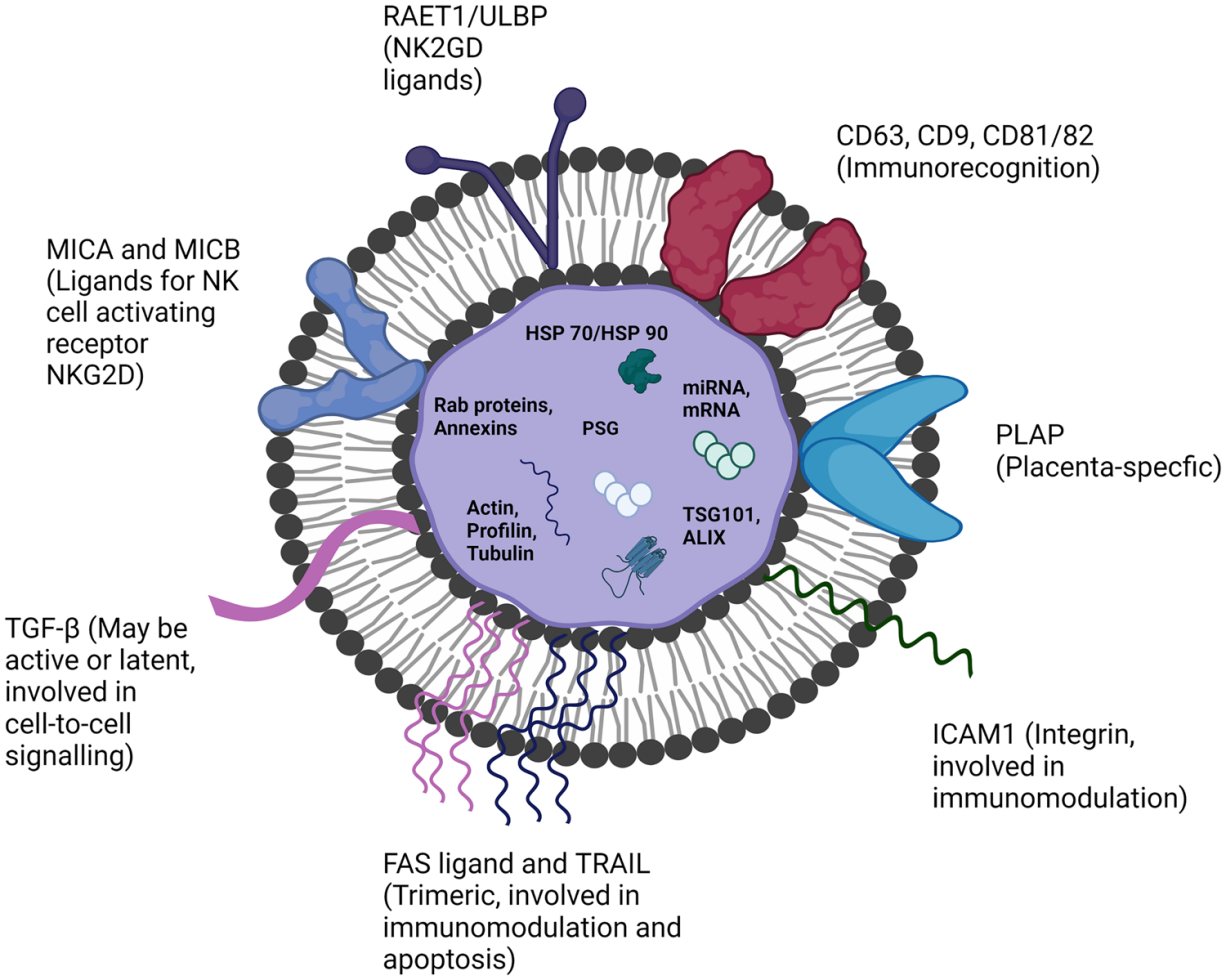
The morphology of a placental exosomes, noting the roles of specific markers which mainly relate to immunomodulatory functions [36, 47], together with cellular signalling and recognition markers [47], which include placenta-specific domains [35]

Placental exosomes are morphologically very similar to exosomes from other mediums such as milk and urine. Their spherical shape and size are retained (30–150 nm) together with their function of cellular signalling [35]. While the typical exosomal markers such as CD63 are retained, placental exosomes also carry the specific enzyme ‘placental-type alkaline phosphatase’, which is indicative of their origin [35]. Further, placental exosomes do not carry the marker molecule MHC–which is typically involved in stimulating T cell proliferation through direct complex binding and results in site-specific immune stimulation [39]. Considering, placental exosomes instead display MHC-related molecules such as MICA/MICB, RAET1, and ULBP1-5 on their outer surface, which are also ligands of NKG2D–an activating NK cell receptor [48]. Placental exosomes have also been shown to express proapoptotic molecules such as the Fas ligand (FasL) and tumour necrosis factor–related apoptosis-inducing ligand (TRAIL), which are involved in proliferative immune responses [49]. A ‘typical’ placental exosome drawing is presented in Fig. 3, based on descriptions of previous research.

**Fig. 4:**
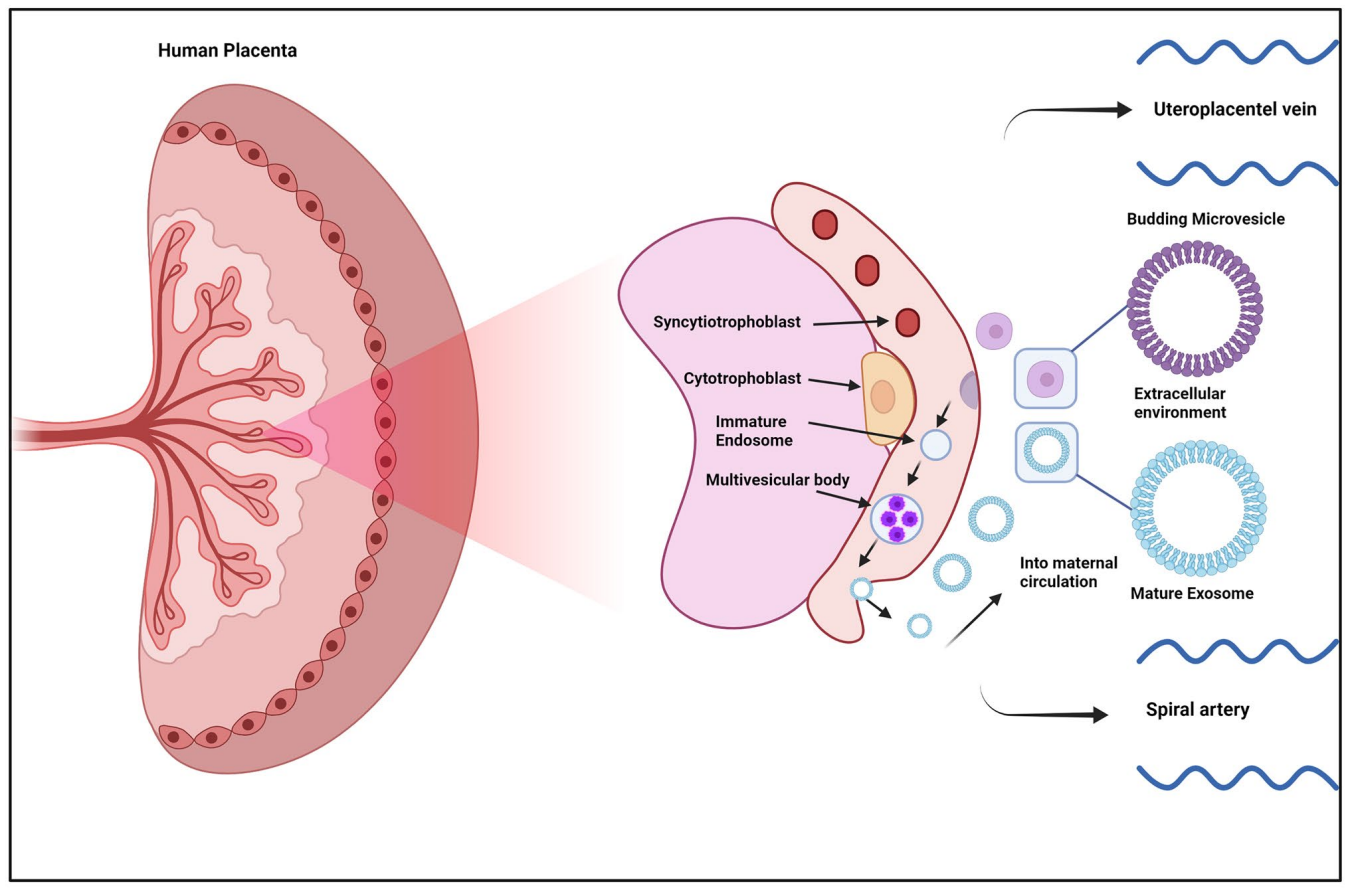
The cellular components of the intervillous space of the chorionic villi, leading on from the umbilical cord and housing the process exosomes maturing from budding microvesicles and eventually being released into the extracellular placental environment to be sent into maternal circulation. The main differences between a budding microvesicle and an exosome are that structures such as proteins and nucleic acids are more pronounced and distinctive in the mature exosome–harbouring the intracellular components necessary for cell-to-cell communication and transport [42, 50]

During pregnancy, the placenta modulates a variety of mechanisms relating to immunotolerance, nutritional balance, inflammatory regulation, and oxygen support, all within the grounds of supporting the foetus [50]. The human placenta secretes a multitude of extracellular vesicles, most of which are derived from the cytotrophoblast, syncytiotrophoblast (STB), and extravillous trophoblast cells [6, 42]. Larger EVs are also secreted by the placenta through the apical membrane of the STB cells for release into the maternal circulation during cell-to-cell communication (Fig. 4), which work together with exosomes to continue the process of ongoing conception [50]. Exosomes become important immunomodulatory messengers during pregnancy–a notable characteristic is their ability to downregulate T cell responses in pregnant women, resulting in less overall inflammation and immune-related reactions at target sites involved in reproductive processes [43]. The downregulation of the T cell immune response assists with appropriate foetal recognition and by extension regulates inflammatory pathways [51].

Similar activity has been described in previous research where placental exosomes have been shown to exert an immunosuppressive effect through the protein syncytin-1 [52]. Through this protein, placental exosomes have a distinguished role in suppressing the production of Th1 cytokines, TNF-α, CXCL10, and IFN-γ [52]. These cytokines are well known for producing pro-inflammatory effects and inducing immune responses [52]. Together with the ability to suppress certain inflammatory cytokines, exosomes assist in the disposal of already circulating cytokines through targeted encapsulation processes, which also assists to dispose of any leftover cytokines following premature immunological stimulation [52]. As Th1 becomes more downregulated during pregnancy, there is a physiological switch to more Th2-type immune responses to avoid any potential damage to the foetus [52]. For example, previous research has shown that an increase in a Th1 immune response and cytokines (TNF-α, CXCL10, and IFN-γ) has been associated with pro-inflammatory pathways and an increased incidence of adverse developmental events and miscarriages [52, 53]. Hence, exosomes which carry syncytin-1 may reduce the occurrence of adverse maternal events and assist in regulating the levels of inflammation during the multiple stages of conception [50–53].

### Exosomes in the foetal development process

The ongoing development of the foetus is highly dependent on feto-maternal communication [37]. As placental development approaches the final stages, the foetal-placental circulatory system continues to act as a pathway for various substituent exchanges, including oxygen, nutrients, hormones, cytokines, and EVs such as exosomes [37]. During the later stages of pregnancy, exosomes derived from chorionic villous tissue have been shown to be more involved in regulating metabolic pathways relating to carbohydrate metabolism and glucose uptake by cells [54]. This was demonstrated through further investigation into exosome samples isolated from patients with gestational diabetes, in which glucose uptake was reduced–indicating a potential role for exosomes in the glucose exchange system [55]. In the later stages of foetal development, exosomes have been found to be involved within immunoregulation and protecting the developing foetus from the innate immune system [56]. In relation, one study found that exosomes can regulate the maternal immune response through expressing the negative immunoregulatory factors PD-L1/L2 [56]. The potential effects of these exosomes point to the downregulation of the maternal immune system through regulating immune functions and vascular development throughout ovulation and general follicular development [56]. Additional exosomal functions may also include modulating naïve cells in concordance with regulatory T cells through binding on the PD-1 receptor of the related immunomodulatory cells [57].

## Exosomes as therapeutic tools in reproductive pathology

Exosomes contain cellular cargo relating to the physiological state of the donor cell–they may be ideal as therapeutic tools for diagnosing and treating complications during reproduction in both males and females [58]. Recent studies have found that the concentration of exosomes in peripheral blood increases over time until the final stages of pregnancy and is further increased before and during relevant pathological complications [58, 59]. Considering this, exosomes may be utilised for the early detection of many pregnancy-related complications, and therefore may assist providing early interventions through a timely and directed approach. An example from recent literature included the use of circulating exosomal miRNA species for the early diagnosis of foetal ventricular septal defects (VSDs) [60]. The study explained that certain exosomal miRNA species (hsa-miR-186-5p, hsa-miR-199a-3p, hsa-miR-146a-5p, hsa-miR-181a-5p, and hsa-miR-3158-3p) were dysregulated in VSD cases from early in pregnancy, signifying that these miRNAs may act as potential biomarkers [60].

Furthermore, it has been reported that placental-derived exosomes can suppress certain immune responses through increasing lymphocyte apoptosis and reducing CD3 expression [61]. It has also been found that exosomes can regulate the NKG2D receptor on NK, CD8(+), and gamma delta T cells, which has resulted in a reduction of cytotoxicity in vitro [61]. The clinical applicability of exosomes can therefore relate to their utilisation to treat a multitude of disorders during pregnancy, including impaired foetal growth and inflammation (refer to Tables 2 and 3 for a summary).

**Table 2:** Exosomes as therapeutic tools in various reproductive pathology

**Reproductive pathology**	**Exosomal sources**	**Methods of isolation**	**Potential biomarker molecule**	**Potential therapeutic outcomes**	**References**
**PCOS**	Serum plasma, follicular fluid, adipose tissue	Ultracentrifugation, differential centrifugation, ultrafiltration, precipitation	miR-373, miR-640, miR-654-5p, hsa-miR-1299, has-miR-6818, miR-145-5p, miR-192, miR-590-3p, miR-27a-5p, DENND1A.V2, CYP11A, -19A, HSD17b1	Possible early detection of PCOS, silencing defective proteins	[62, 66–71]
**Reproductive inflammation**	Serum plasma, placental fluid, amniotic fluid	Differential centrifugation, ultrafiltration, SEC	hsa-miR-126-3p, hsa-miR-23a-3p, COX-2, GMCSF, IL-6, IL-8	Assist in early detection of inflammation, may help in detecting exact area and cause	[34, 72–74]
**Endometriosis**	Serum plasma, follicular fluid, endometrial stromal cells	Ultracentrifugation, differential centrifugation	miR-134-5p, miR-197-5p, miR-22-3p, miR-320a, miR-494-3p, miR-939-5p, PRDX1, H2A type 2-C, ANXA2, ITIH4, Tα	Easier and faster diagnostics, leading to quicker therapeutics	[75–78]
**POF**	Bone marrow–derived mesenchymal stem cells, granulosa cells	Ultracentrifugation, differential centrifugation	miR-144-5p, miR-127-5p, YY2, CP, CC3, fibrinogen, SHBG	Easier and faster diagnostics, avoid long-term ovarian damage	[62, 66–68]
**GDM**	Serum plasma, urine, blood from the umbilical vein	Ultracentrifugation, silicon carbide ultrafiltration	miR-516-5p, miR-517-3p, miR-518-5p, miR-222-3p, miR-16-5p, miR-125b, miR-144, S100A9, DAMP	Quicker therapeutics, may assist in avoiding long-term complications and early-onset type 2 diabetes mellitus, protect the growing foetus from complications	[69–72]
**Pre-eclampsia**	Maternal circulation blood samples, HEK293T cell line	Ultracentrifugation, differential centrifugation, SEC	miR-153, miR325-3p, miR122-5p, miR2605-3p, miR2113, miR-374c-5p, miR505-3p, versican, biglycan, PZP	Pre-emptive diagnostics, protect the mother and foetus from ongoing complications	[34, 73–76]

**Table 3:** The different reproductive disorders in relation to exosomal isolation, pathogenesis, and limitations

**Reproductive disorder**	**Exosomal isolation method**	**Mechanism of pathogenesis**	**Limitations of related research**	**References**
**Polycystic ovarian syndrome**	Serum filtration through 0.22-μM filters, membrane-based affinity binding, ultracentrifugation steps.	Small RNA sequences in follicular fluid, altered miRNA expression, driver genes.	Low patient study number.	[62, 66, 67, 76, 78]
**Reproductive inflammation**	Ultracentrifugation, differential centrifugation.	Parturition through foetal endocrine signalling–immune driven, preterm premature membrane rupture, inflammatory cytokines.	Unknown paracrine mediators.	[34, 72, 73]
**Endometriosis**	Ultracentrifugation, differential centrifugation.	Inappropriate miRNA-related proliferation, growth, differentiation, and apoptosis.	Limited patient samples and quantification of miRNA species, including validation.	[75, 76]
**Premature ovarian failure**	Ultracentrifugation, differential centrifugation.	Enzyme autoimmunity, gene-driven miRNA overexpression, drug-induced, previous infections.	miRNA characterisation extent, animal models may be inaccurate when transferred to human disease specifications.	[79–82, 84]
**Gestational diabetes mellitus**	Differential centrifugation, spin column chromatography.	Genetic gene mutation, β-cell dysfunction, miRNA up/downregulation, gene dysregulation causing miRNA species imbalances.	Not suitable for larger molecules, damage to the exosomal wall is often irreversible, may disrupt the cargo.	[85–88]
**Pre-eclampsia**	Differential centrifugation.	Hypoxia-inducible factor 1-alpha, miRNA up/downregulation.	Rigour in assay utilisation, limited patient numbers, detection, and quantification of exosomal miRNA.	[74, 90–94]

The reproductive cycle is a complicated mix of highly regulated processes, meaning that problems may occur relating to immunological function, cellular signalling, and nutrition malabsorption–thus, irregular placental and foetal development may ensue [62]. As previously mentioned, exosomes can mediate cell-to-cell communication and carry different cargo based upon the physiological state of the donor cell; this means that specific cargos such as miRNA and proteins may be useful in understanding the pathology behind reproductive disorders [38, 39, 58]. Exosomal function in obstetric pathology can be extended to syndromes such as PCOS, acute or chronic inflammation, endometriosis, POF, GDM, and pre-eclampsia [52]. Furthermore, the relevant pathology also becomes important when discussing male fertility/infertility as to how exosomes contribute to the progression of various functions such as sperm production and maturation, and therefore assist in healthy reproduction, as discussed below [63–65].

### Exosomal functions in male reproductive pathology

Although this review is mainly focussed on reproductive pathology within females, it is important to highlight certain aspects surrounding exosomes in the male reproductive system. Exosomes play a considerable role in the male reproductive system, more specifically in relation to fertility and infertility, being highly involved in sperm maturation, acrosome reactions, capacitation, and fertilisation [63]. These exosomes have been found to originate from within the prostate (prostasomes), testis, seminal fluid, and epididymosomes [63]. Due to their supportive role in various seminal functions, they have been associated with the progression of normal reproductive cycles and are implicated within the transportation of various regulatory proteins and nucleotides [63].

Recently, a study focussed on the exosomal profiles of azoospermia patient semen samples and found that several types of RNA (miRNA, piRNA Y RNA, rRNA, and tRNA) were present in the related exosomes, with miRNA displaying the most differential profile compared to other bodily fluids [63]. Considering, the study found that many miRNA levels were dysregulated in seminal plasma exosomes, including species which were germ-cell specific [63]. Importantly, a high predictive accuracy was found in certain RNA species (miR-205-5p, miR-31-5p, and germ cell piR-58527) with a diagnostic efficiency of AUC > 0.95 specifically for miR-31-5p and a related high level of sensitivity and specificity [63]. While more research needs to be established, miRNA-based exosomal profiles may assist in building an efficacious diagnostic model for azoospermia and related possible causes.

The possibility of diagnostic models has been found in exosome-associated proteins, which are important for the maturation of spermatozoa and can thus also be implicated as potential biomarkers for male infertility [64]. One study described that exosomal proteins annexin A2 (ANXA2), semenogelin-1 (SEMG1), transferrin (TF), and kinesin-1 heavy chain (KIF5B) were dysregulated in male patients with varicocele–a condition characterised by the formation of varicose veins within the scrotum which can often lead to infertility [64]. As these proteins are variable in their expression, it may be possible to utilise them as biomarkers to detect early signs of infertility in males [64].

Another similar study compared seminal exosomal protein levels in fertile versus infertile men and distinguished that within the infertility group, many proteins were differentially expressed, including the upregulation of ANXA2 and the downregulation of KIF5B [65]. The same study found that 47 seminal plasma proteins were dysregulated in unilateral varicocele patients in comparison to controls–it was further deducted that dysregulated proteins were correlated to androgen receptors YB1 and NRF2 [65]. Overall, exosome-associated proteins may be useful in the early diagnostics of male infertility and may also point towards the relevant pathology involved within the progression of reproductive disorders [64, 65].

### Exosomal functions in female reproductive pathology

In relation to female reproduction, many of the pathways involved in the pathological progression of reproductive disorders are connected to exosomes and their cargo, such as miRNA species and proteins (refer to Table 2). While there has been relevant research in this field, many studies have had various limitations relating to smaller sample sizes, and narrow or inconclusive results, with much research not being replicable to an acceptable standard. Further, research gaps can be identified relating to various exosomal biomarkers and how these may be used in a clinical setting, such as for diagnostic or interventional purposes. Hence, it becomes important to further explore as to how exosomes may be of viable clinical applicability when discussing reproductive pathology [38, 39].

#### Exosomes in polycystic ovarian syndrome

PCOS is a common reproductive endocrine disorder affecting approximately 8–10% of women of child-bearing age in which pathological changes occur in relation to abnormal follicular granulosa cell proliferation, abnormal apoptosis, and hyperandrogenism [62, 66]. PCOS can be phenotypically characterised through affected individuals experiencing symptoms relating to hair loss, hair overgrowth, obesity, amenorrhea, and menstrual irregularities [66]. As exosomes are involved in follicular development through cellular signalling and communication, specific exosomal biomarkers may indicate towards the progression of PCOS, which ultimately may lead to an early intervention. Recent studies have found that cargo from follicle-derived exosomes includes various PCOS-specific miRNAs such as miR-373, miR-640, and miR-654-5p, with newer biomarkers being hsa-miR-1299, hsa-miR-6818-5p hsa-miR-192-5p, and hsa-miR-145-5p [66, 67]. These specific miRNA biomarkers have been found to be overexpressed in PCOS patients and may possibly be used as diagnostic tools [67].

Furthermore, another recent study found that serum exosomes from PCOS patients expressed highly elevated levels of miR-590-3p and miR-27a-5p [62]. Functionally, miR-27a-5p in PCOS-derived exosomes was found to promote proliferation and migration in endometrial cancers, which may be linked with the progression of PCOS through the driver gene SMAD4 [62]. This pathway was found to occur through the targeting of the SMAD4 gene by miR-27a-5p, in which the gene is upregulated, and migration and proliferation are promoted [62]. Conversely, one study found that exosomal miR-323-3p derived from mesenchymal stem cells both promoted proliferation and inhibited apoptosis in PCOS, which resulted in the condition somewhat improving [68]. Additionally, another recent study explained that PCOS follicular fluid–derived exosomes carrying miR-424-5p upregulated granulosa cell senescence through the direct targeting of the CDCA4 gene, which resulted in downregulated cell proliferation [69].

Research has also found that the DENND1A.V2 protein was higher in PCOS theca cells compared to that in controls, which may have an indirect downstream effect on insulin and luteinising hormone through the RAB5B system [70]. Similarly, one study indicated that significantly higher levels of mRNA expression relating to the proteins CYP11A, CYP19A, and HSD17b1 were found in follicular fluid of PCOS patients compared to study controls [71]. The dysregulation of such constituents can also point to the strategy of loading these particles into exosomes to act as therapeutic cargo for targeted delivery, where specific types of molecules such as miRNA or proteins could mediate dysregulated pathways. Thus, the miRNA exosomal markers may have the potential to provide a basis for the early diagnosis of PCOS and understanding precipitating factors which may also promote the development of endometrial cancer from PCOS [62, 66–71].

#### Exosomes in reproductive inflammation

Exosomes carry inflammatory mediators which differ among cellular pathways depending on their site of origin and can signify the unique properties and disease state of their original environment [72]. One study explains that exosomal inflammatory mediators were observed in mice in gestation days E5 to E19 and that increased inflammatory markers can be responsible for causing early inflammatory activation in maternal gestational cells [72]. Important inflammatory markers in reproduction are cyclo-oxyenase-2 (COX-2), granulocyte–macrophage colony-stimulating factor (GMCSF), interleukin 6 (IL-6), and interleukin-8 (IL-8) [73]. These markers have the potential to provide information on the state of the host cell and how some inflammatory processes may progress within the reproductive cycle [73]. Research has found that the pro-inflammatory cytokines GMCSF, IL-6, and IL-8 were abundant in exosomes which were exposed to increased levels of oxidative stress (OS), and in turn contributed to a state of inflammation in various tissues [73]. Additionally, foetal-derived exosomes were found to exhibit differential characteristics and were distinct in promoting an inflammatory state in uterine cells, which was associated with the initial development of parturition [73].

Exosomes may have modulatory roles in various inflammatory pathways, and potentially be involved in the up- and downregulation of pro-inflammatory cytokines. Adding to this, a recent study explained that exosomes derived from amniotic fluid can project the current inflammatory status of the uterine environment through their specific miRNA and protein contents [74]. Furthermore, miRNA biomarkers are of particular interest in distinguishing maternal systemic inflammation [34], for example, the upregulation of hsa-miR-126-3p and hsa-miR-23a-3p, which are both involved in pathways relating to vascular cell adhesion molecule 1 (VCAM1) inhibition, limiting leukocyte cell adhesion, and targeting ATG12-mediated autophagy [34]. Such exosomal biomarkers could possibly indicate the development of abnormal pregnancies and provide specific information about uterine health, including any imminent risks and predisposing factors contributing to a potential miscarriage [74].

#### Exosomes in endometriosis

Endometriosis can be defined as the presence of endometrial tissue around the outside of the uterine cavity and affects approximately 10–15% of women of reproductive age [75]. The main symptoms are presented as pelvic pain, infertility, heavy bleeding, and ovulatory pain [75]. Exosome-derived miRNA species may be considered potential diagnostic biomarkers for various reproductive disorders, including endometriosis. Research has found that exosomes derived from follicular fluid have shown differences in the contents of their cargo between control and PCOS patients, specifically relating to miRNA species [76]. It was explained that the differences in exosomal cargo could alter processes relating to the development and progression of endometriosis, indicating that exosomal cargo may be involved in regulatory pathways [76]. Relatedly, a recent study explored the use of endogenous exosomal miRNA for the early diagnosis of endometriosis through attempting to identify multiple dysregulated miRNAs in serum exosomes derived from patients [75]. Some notable targets were namely miR-134-5p, miR-197-5p, miR-22-3p, miR-320a, miR-494-3p, and miR-939-5p–the main miRNA biomarkers which were highly upregulated in endometriosis patients were found to be miR-22-3p and miR-320a [75]. These miRNA targets were found to have a high specificity towards the progression of endometriosis and could potentially increase the diagnostic sensitivity relating to screening for and treating endometriosis early and effectively [75].

Similarly, another recent study found various exosomal biomarkers relating to the progression of endometriosis [77]. The notable biomarkers circular RNA_0026129, miRNA-15a-5p, and the genomic marker ATP6V1A were highly related to the endometriosis-associated exosomal competing endogenous RNA network [77]. Overall, the study found these markers to be differentially expressed between endometriosis patients and related healthy controls, indicating reliability in sequencing and their possible use as specific biomarkers for diagnosis or targeted treatment [63]. Hence, these may indicate the different stages and processes in the progression and development of endometriosis during pregnancy. Relatedly, another recent study described dysregulated proteins in endometriosis patients compared to healthy control patients, of which includes PRDX1, H2A type 2-C, ANXA2, ITIH4, and the tubulin α-chain (Tα) [78]. These proteins may have endometriosis-specific roles and in which case would assist with early detection of related pathology, although further research would be required to confirm uniqueness [78].

#### Exosomes in premature ovarian failure

Premature ovarian failure (POF) is a disease of women’s reproductive health which results in the premature cessation of ovarian function before the age of 40, and its prevalence is largely genetically linked [79]. Individuals will typically present with symptoms of reduced oestrogen levels, amenorrhea, infertility, reduced mature follicles, and high gonadotropin levels [79]. POF accounts for approximately 1% of infertility occurrences in females, and as fertility is key to reproduction, it becomes pertinent to screen for POF as early as possible [80]. Overall, recent literature has stated that the follicle-derived exosomal miRNA component miR-144-5p has been used to identify and treat chemotherapy-induced ovarian failure in animal models, indicating promise towards a potential POF biomarker [81].

Furthermore, another recent study found that the transcription factor Yin Yang 2 (YY2) is significantly reduced in patients with POF [82]. The study focused on exosomes derived from peripheral blood from patients with POF and denoted a positive correlation between progesterone/oestradiol levels and YY2, as these levels are usually diminished in patients with POF [82]. Hence, YY2 was found to be related with fluctuating hormonal levels during disease progression in POF, making it a possible target for early diagnostics and therapeutics [82]. Additionally, another recent study found that exosomal miR-127-5p, a miRNA involved in downregulation pathway of DNA repair mechanisms, was overexpressed in patients with POF [83]. Furthermore, a similar study noted the presence of multiple up/downregulated proteins in patients with POF [84]. These included proteins involved in the reproductive process such as ceruloplasmin (CP), complement C3 (CC3), fibrinogen, and sex hormone binding globulin (SHBG) [84]. These protein biomarkers were described to be increased twofold within POF patients compared to the control group [84]. Their relevance can be linked to specific functions, such as CP being important for transporting copper throughout the body, especially during pregnancy [84]. Relatedly, CC3 is involved in the complement system, which is a part of the regulation cycle of immune system and phagocytosis [84]. Finally, fibrinogen plays a pivotal role in clotting factors to stop bleeding and SHBG is attached to androgens and estrogens and is usually increased in postmenopausal women [84]. Hence, dysregulation of these proteins may help in understanding a variety of disease factors before, during, and after POF.

#### Exosomes in gestational diabetes mellitus

Gestational diabetes mellitus (GDM) affects roughly 14% of worldwide pregnancies and is a complication in which patients without a previous history of diabetes begin to develop clinically significant and chronic levels of hyperglycaemia during gestation [85]. The development of GDM is linked to the impairment of glucose tolerance in relation to pancreatic β-cell dysfunction and is usually more common in women with pre-existing risk factors [85]. Such can include being overweight, previous family history of diabetes, and an advanced maternal age [85]. Exosomes may improve both early diagnostics assist in tailoring therapy for the management of GDM, which in turn may act as a type of prevention and treatment for both the foetus and the mother.

A recent study explored potential exosomal miRNA biomarkers which were downregulated in GDM and during the 3rd trimester of gestation (miR‑516‑5p, miR‑517‑3p, miR‑518‑5p, miR‑222‑3p, and miR‑16‑5p) [86]. The downregulated exosomal miRNAs were linked to various metabolic pathways associated with the development and progression of GDM. This indicates that exosomes and their cargo may be pivotal in cellular pathways relating to inflammation, energy production, and insulin mobilisation [86]. Furthermore, the cellular mechanisms of the related miRNA correspond to pathways in stress responses and variations in circulating blood glucose levels [86]. Another recent study found that circulating exosome release was higher in GDM patients compared to non-GDM patients, and that exosomal miRNA may affect pathways relating to lipid metabolism, glucagon signalling, and glucose homeostasis [87]. The research suggests that exosomes which express specific contents could modulate various metabolic pathways and alter processes in which normal metabolism may be dysregulated through the stages of pregnancy.

Specific miRNA species could possibly be used as both biomarkers and therapeutic targets and further indicate as to which metabolic pathways may be dysregulated before and during GDM [87]. Another study which focused on exploring different potential exosomal miRNA biomarkers found that miR-125b was consistently downregulated in GDM while miR-144 was found to be consistently upregulated [88]. The authors further performed AUC models for both miR-125b and miR-144 and obtained results of 0.898 and 0.875 respectively, indicating favourable diagnostics [88]. These findings detail the dysregulation of miR-125b and miR-144 within GDM and outline their potential use as diagnostic tools. Additionally, another research study explained the presence of protein biomarkers through their dysregulation within GDM patient samples, specifically S100 calcium binding protein A9 (S100A9) and damage associated molecular patterns, which are involved in cell cycle progression/differentiation and the innate immune response system, respectively [89]. The study found that a more specific increase in S100A9 protein numbers correlated to maternal obesity in GDM patients and increased the chances of macrosomia in newborns [89]. Hence, a mixture of such miRNA and protein biomarkers may be more efficient in determining treatment options in GDP patients and providing better long-term patient outcomes [89].

#### Exosomes in pre-eclampsia

Pre-eclampsia can be a common and serious complication during pregnancy, in which 5–8% of pregnancies are affected and the exact pathological cause remains an unknown area, with higher incidences in at-risk populations, such as smoking, obesity, and a family history of hypertension [90]. The disorder is characterised by symptoms such as hypertension and multiple organ injury stemming from placental malperfusion in which various disease-promoting factors are released into maternal circulation [90]. As no medication has been shown to completely alleviate the progression of the condition, early diagnostics may help with the initial management and timing during the pregnancy to optimise foetal and maternal outcomes. Considering, exosomes may help in understanding the pathologies involved in pre-eclampsia and how this condition may develop, including a multitude of other reproductive disorders. A recent study showed that isolated exosomes from patients with pre-eclampsia contain several miRNA makers specific to the progression of pre-eclampsia [91]. The identified exosomal miRNA species included miR-153 and miR-325-3p, which were both significantly upregulated in pre-eclampsia [91]. Literature has found both miR-153 and miR-325-3p to be associated with reduced tube formation in primary human umbilical vein endothelial cells and endothelial cell dysfunction, respectively [92, 93]. Considering the high amount of upregulation of these miRNA species, they may be utilised as potential biomarkers and provide more information regarding pre-eclampsia during the different stages of pregnancy [91].

**Fig. 5:**
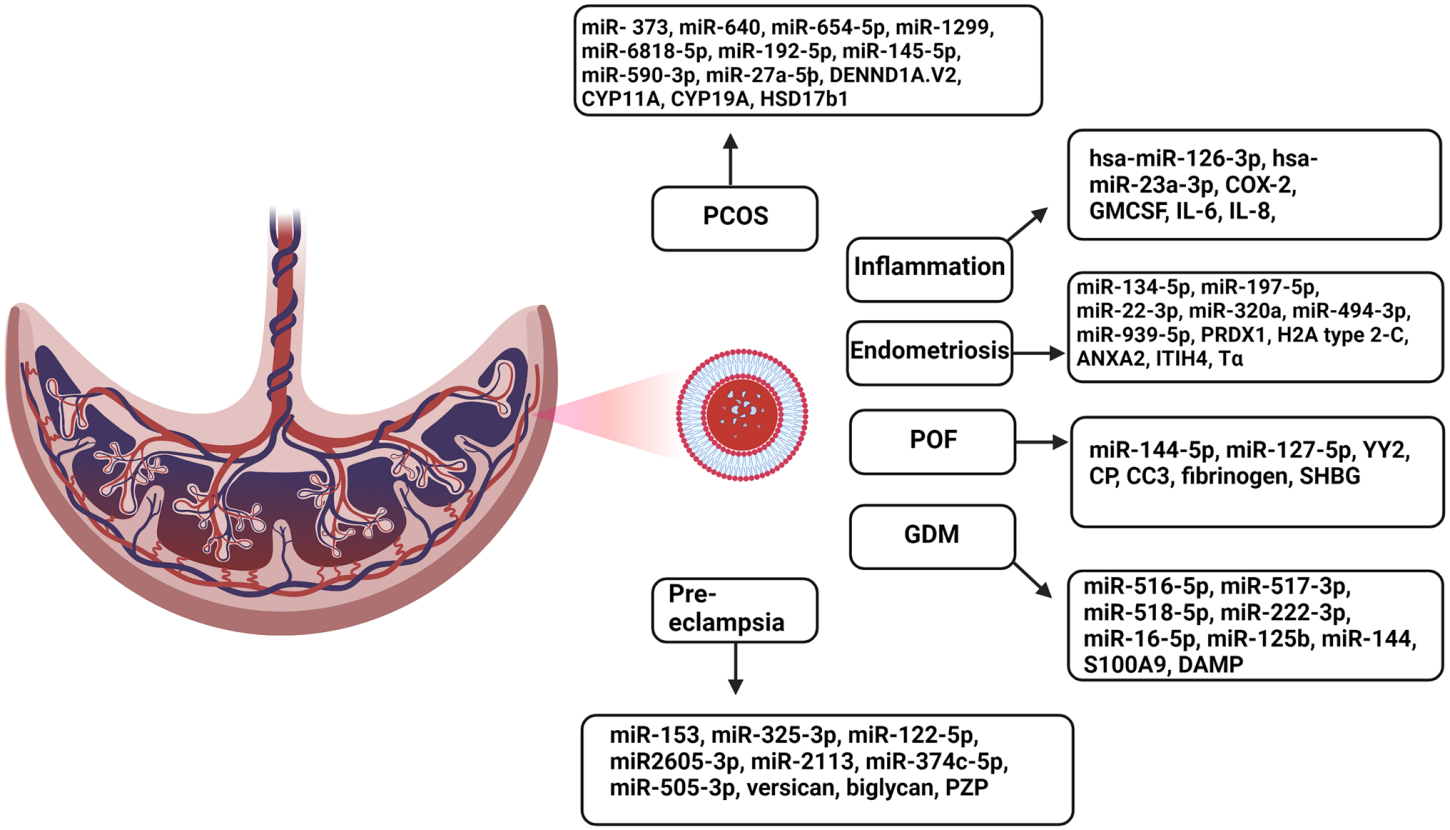
The exosome within the structure and vessels of the placenta, including the various exosomal markers which may indicate the early diagnosis of different reproductive diseases. This summary includes–polycystic ovarian syndrome (PCOS) [62, 66–69, 76, 78], uterine and reproductive inflammation [34, 73, 74], endometriosis [75, 76], premature ovarian failure (POF) [79–82, 84], gestation diabetes mellitus (GDM) [85–88], and pre-eclampsia [74, 90–94]. These exosomal markers are possible milestones of the pathological responses which are undergone in these reproductive diseases and indicates that early diagnosis may indeed be a possibility

Similarly, another study explored related miRNA exosomal biomarkers which could describe the stages of pre-eclampsia relating to late-onset and early-onset, such examples included miR-122-5p, miR-3605-3p, miR-2113, miR-374c-5p, and miR-505-3p, which confirms that a multitude of miRNA species may be involved in the pathogenesis of pre-eclampsia (see Fig. 5) [74]. The relevance of miRNA species is related to the variation between patients with and without pre-eclampsia–however, further research is required to ascertain exact pathogenesis [74]. A recent study looking at therapeutic targets found that miRNA (miR-18b-3p) obtained from human umbilical cord mesenchymal stem cell–derived exosomes inhibits the development of pre-eclampsia through targeting the leptin protein [94]. The study found that inhibition of leptin through miR-18b-3p lowered systolic blood pressure and proteinuria in pre-eclampsia rat models. With the result of leptin inhibition, it would be possible to load this miRNA cargo into exosomes for targeted delivery into patients who have or are at risk of pre-eclampsia and gestation hypertensive issues (refer to Table 3). Furthermore, one study found that exosomal PLAP levels was increased in women who developed pre-eclampsia, which could potentially differentiate between exosomal content useful in determining biomarkers [95]. Another recent study identified increased levels in pre-eclampsia-associated proteins as being the glycocalyx-associated proteins, versican and biglycan [96]. Both proteins have been implicated in endothelial dysfunction and eventually pre-eclampsia [96]. The study also found that women with pre-eclampsia displayed reduced levels of the pregnancy zone protein (PZP), which is involved in the inhibition of misfolded protein aggregates [96]. The reduced PZP levels may be associated with a high incidence of protein aggregates in patients with pre-eclampsia [96]. However, other barriers exist towards therapeutic progression–such as efficient exosomal cargo loading, targeted delivery, and the translation of exosome therapeutics from the laboratory setting to a clinical setting. These barriers must be overcome so factors such as cargo loading, and delivery may be efficiently and safely implemented.

## Exosomal isolation and loading methods

Exosomes are important mediators for cell-to-cell communication, especially in disease pathology. It becomes pivotal to investigate exosomes as potential therapeutic delivery vehicles for medications, miRNA/siRNA, and other proteins or compounds [97]. The therapeutic suitability of exosomes relates to their ability to pass certain biological or physical barriers like the BBB or the placenta, while having an excellent immunogenicity profile–resulting in fewer immune-related reactions such as T cell foreign body destruction. Furthermore, exosomes can protect cargo from enzymatic degradation due to their phospholipid bilayer, alongside increasing drug bioavailability and reducing rapid drug clearance through their mononuclear phagocyte system [98].

As the state of pregnancy is one in which many bodily processes are altered to accommodate a growing foetus, it becomes difficult to effectively diagnose and treat many of the common complications during this period [79]. Currently, various limitations exist in diagnosing and treating complication within pregnancy, some of which include a lack of diagnostic tools, limited available treatments, and limited medications which may be safely used [99]. For example, inflammatory and autoimmune conditions can be relatively common during and after pregnancy. A significant concern in treatment is the safe use of medications which reduces inflammation such as prednisolone, which is prone to potentially cause off-target effects and affect the foetus–including increasing the risk of GDM and pre-eclampsia [99]. Exosomes have the capability to overcome medication side effects through the encapsulation of therapeutic molecules and targeted delivery to the intended site of action [99].

Additionally, pre-eclampsia is another potential complication during pregnancy in which diagnosis is not always clear and medications have not been found to greatly influence disease progression in a positive manner [90]. However, previous clinical trials have indicated that more consistent and accurate diagnostic and prognostic tools may improve patient outcomes [90]. Thus, exosomes may hold the answer towards improving diagnostics and treatment using miRNA biomarkers and the safe delivery of anti-hypertensive medications. Through this, it could potentially allow for early diagnostics and interventions, overall reducing maternal and foetal risks and increasing positive patient outcomes.

**Fig. 6:**
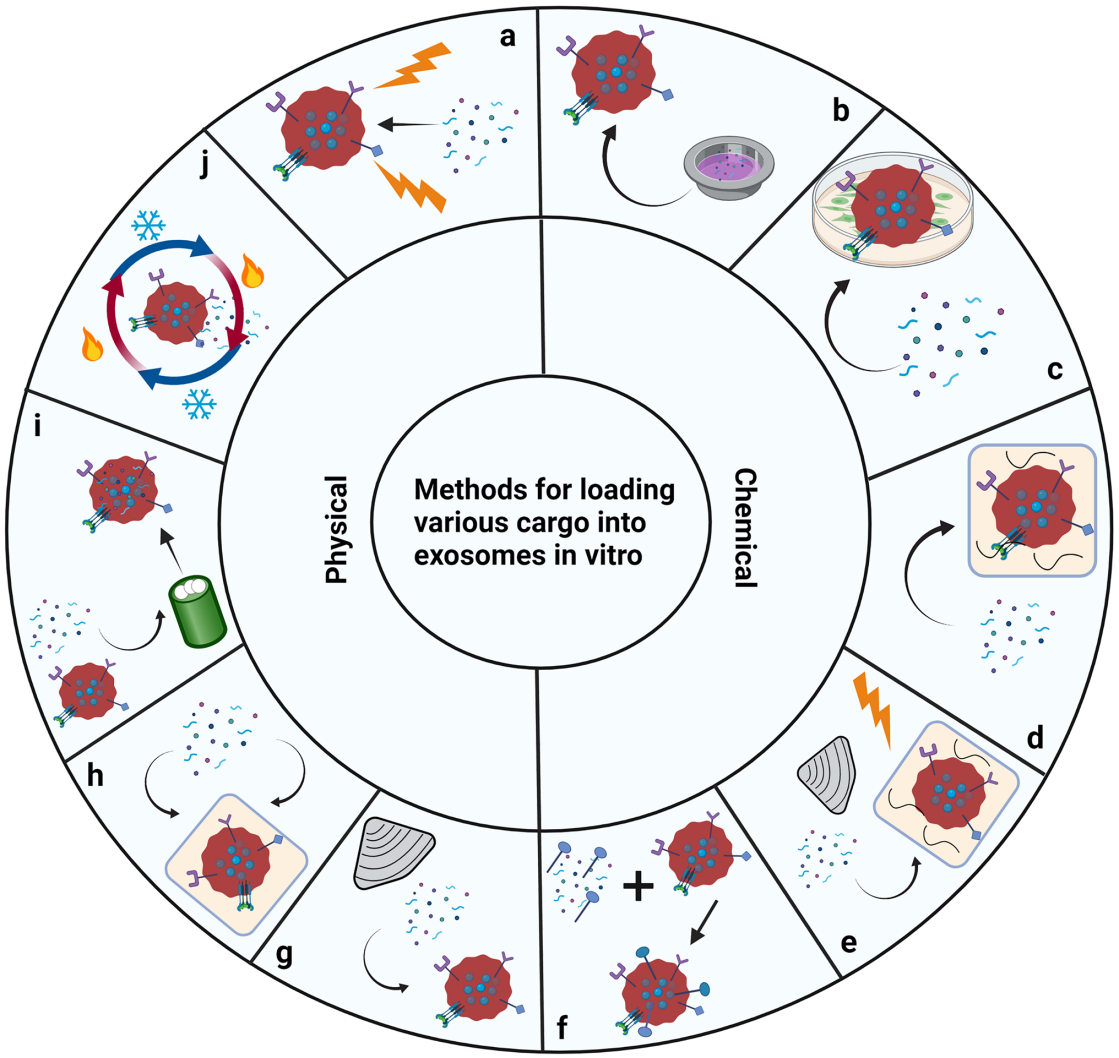
The various methods for loading cargo into exosomes, including **a** electroporation, a physical method involving using electrical current to increase micropores in exosomes; **b** transfection, using specific reagents to load plasmids for the expression of desired cargo; **c** utilising a membrane permeabiliser (such as saponin) to increase the surface permeability of the exosome; **d** utilising a membrane permeabiliser with incubation to create larger pores; **e** utilising a membrane permeabiliser with electroporation or sonication, creating larger pores; **f** click chemistry, using copper-catalysed azide alkyne cycloaddition to form strong bond between particles and thereby help by chaperoning the desired cargo into the exosome; **g** sonication, a physical method in which sounds waves are utilised to create micropores in the exosomal surface to assist with particle loading; **h** incubation, incubating exosomes with desired cargo for the creation of a concentration gradient to assist with loading; **i** extrusion, another physical method, using an extruder to squeeze particle into exosomes; and **j** freeze–thaw, another physical method, using multiple cycles of freezing and thawing to mechanically push particles into exosomes

**Fig. 7:**
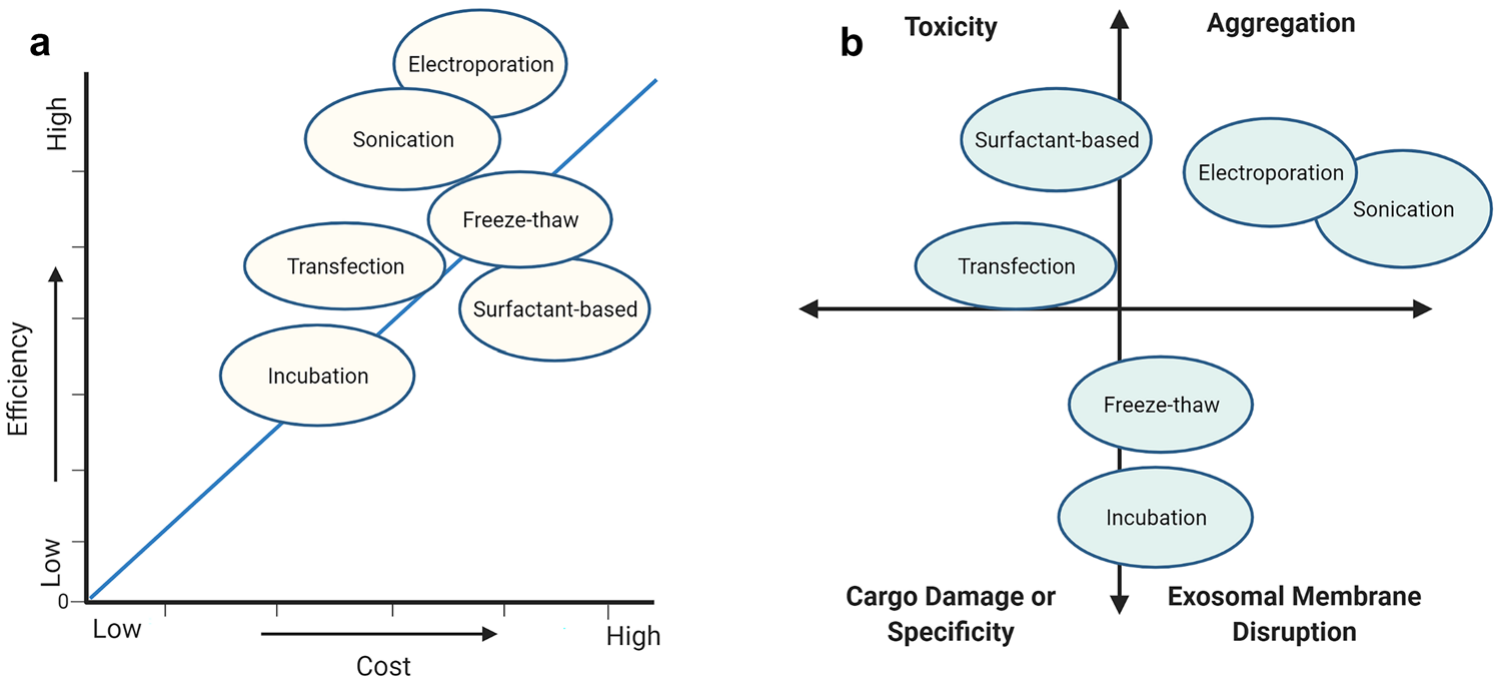
The various factors influencing exosome cargo loading and targeted drug delivery in clinical applicability. **a** Line cluster graph showing the different relative costs and efficiencies of exosome loading techniques. As can be seen, electroporation is efficient for loading exosomes and is relatively inexpensive, alongside permeabiliser-assisted loading, and to an extent, extrusion. Passive exosome loading remains mixed in its efficiency, although the cost in inexpensive if the materials are available. **b** Cluster vector chart displaying common issues which may arise specific to the loading method which is being utilised–adapted and based on previous research [81, 82]

Exosome loading becomes highly relevant when discussing the therapeutic potential in pregnancy and the possibility for targeted medication delivery. However, this is an area which needs further research to develop new methods and improve on already established methods. Currently, many methods exist for exosomal isolation and cargo loading, all of which differ in aspects relating to loading efficiency, constituent viability, difficulty, cost, and appropriateness in terms of the specific cargo (see Figs. 6 and 7) [100–102]. An important factor to consider is the loading method used for specific cargo. Method selection becomes important because certain molecules will react very differently when exposed to conditions such as electrical current, sound waves, temperature fluctuations, mechanical force, and other chemical compounds. Overall, it becomes important to first understand the various conditions of exosomal isolation and loading and to further explore how improvements and optimisations can be made for loading therapeutic cargo, such as drug molecules [100].

### Exosome isolation methods

Various methods exist for isolating exosomes, and vary on aspects relating to yield, purity, equipment, cost, and amount of time required to perform the specific isolation method [103–105]. As exosomes differ in characteristics such as size, source, content, and presence within different mediums, isolation can be challenging and variable as many techniques cannot fully separate exosomes from other counterparts related to nanovesicles [103]. Such challenges often manifest through results of low exosomal yield, purity, or damaged content [103]. As explained by Théry et al. and as per guidelines by the International Society of Intracellular Vesicles, exosome isolation remains an integral part of the therapeutic development process and includes various isolation methods which are selected depending on exosomal applications [106]. These include ultracentrifugation, differential centrifugation, size-exclusion chromatography (SEC), immunoaffinity isolation, polyethylene polymer precipitation, microfluidics, and ultrafiltration (refer to Table 4).

**Table 4:** The different exosomal isolation methods most used in laboratory settings

**Exosomal isolation method**	**Methodological reasoning**	**Advantages**	**Disadvantages**	**References**
**Ultracentrifugation**	Separation of various sized particles, being able to isolate through density and force.	Usually, the gold standard for exosome isolation, easy to perform, inexpensive, results in higher exosome purity.	Highly time-dependent process, multiple spin cycles may be required, can damage exosomes, generally produces lower exosomal yields.	[103–105]
**Differential centrifugation**	Particle separation through incremental increases of centrifugation speed, starting from lower to higher speeds.	May separate different particles more efficiently due to stepwise speed increases, easy to perform, inexpensive, high purity.	Multiple spins may damage exosomes, generally lower yields, spin number for isolation is not a set amount.	[107, 108]
**Size-exclusion chromatography**	Particles in a solution are separated by their size and molecular weight through a gel-based medium.	Fairly simple to perform, inexpensive, can separate large and small molecules efficiently, minimal loss of volume, and generally high in exosome purity.	Depending on the specific columns, exosomal loss may be an issue, more common for sample contamination, special equipment required for columns, may be expensive.	[109–111]
**Immunoaffinity**	Relies on the interactions between antibodies and various EV membrane proteins to isolate specific EVs.	Purity is generally high as specificity for EV type is high, can also produce high yields.	May be expensive compared to other methods, contamination may be an issue, highly dependent on requiring viable samples for isolation, time consuming.	[112, 113]
**Polyethylene polymer precipitation**	Based on the idea of utilising a hydrophilic polymer to lower the solubility of EVs, hence inducing their precipitation and easy collection.	Usually produces high yields, simple method to perform, flexibility in desired volume usage.	May damage exosomes to an extent, contamination may be an issue, aggregation is common.	[114–116]
**Microfluidics**	Pre-enrichment method usually used for EVs from blood samples of cell cultures–utilises antibodies on microfluidic surfaces to attract desired EV.	Often results in high exosome purity and yield, leads to fast isolations, versatility in EV sorting, high sensitivity in sorting EVs.	May be expensive to perform, more complicated than other more established methods, aggregation and contamination can be issues.	[117–119]
**Ultrafiltration**	Utilisation of a nanomembrane to separate various molecules based upon their molecular weight.	Simple method to perform, flexibility in desired volume, destroys possible bacteria contaminants.	Can be an issue separating low molecular weight species, exosomal loss may occur, membrane clogging can be common.	[120, 121]

#### Ultracentrifugation

Exosomal isolation through ultracentrifugation is based on the separation of different-sized particles through high centrifugal forces and has often been a preferred and simple method [103, 104]. Although ultracentrifugation is straightforward and inexpensive if the equipment is readily available, it is time-consuming and commonly results in variable exosomal yields, albeit the high level of purity within exosomal samples [103, 104]. Relatedly, it was found that ultrafiltration provided a larger yield of exosomes, which can be translated to higher loading efficiencies where many exosomes are readily available [103, 104].

#### Differential centrifugation

Similar to ultracentrifugation, differential centrifugation is based on separating particles through centrifugal forces related to the particle sedimentation rate [107]. This process relies on a stepwise procedure wherein centrifugal force is increased in varying increments, thereby separating particles layer by layer [108]. Lower speeds are used to separate larger particles while higher speeds are used to separate smaller particles, hence the increase in speed over time [108]. Overall, this method may provide high purity exosomes and less general debris due to its rigorous nature. However, exosomal yield may be hindered as multiple spin cycles may exclude exosomes to an extent and damage existing exosomes [107, 108].

#### Size-exclusion chromatography

SEC is an isolation method which works by separating molecules by their size and weight (for heavier particles) [109]. This method is useful for separating larger molecules from smaller molecules and generally results in high exosomal yields and variable purity with a minimal loss in overall volume [110]. When utilising SEC, it becomes important to use a reliable column for the adequate separation of different molecules by their relative size and weight [110]. If a suitable column is not present or if clogging occurs, the sample may become contaminated, or exosomal yield and purity may be insufficient [109]. Hence, while this method is useful and easy to perform, it bears a low threshold for error and sample recovery, should the equipment not be appropriate or properly calibrated [109–111].

#### Immunoaffinity

Immunoaffinity-based exosome isolation relies on the interactions between specific membrane proteins and binding antibodies [112]. Exosomes can be isolated by using antibodies which are specific to exosomal proteins, and through receptor and ligand interactions [112]. Immunoaffinity isolation provides higher exosome purity although variable yields and can be an expensive and time-consuming method to complete, it is not usually the isolation method of choice [112, 113]. Since immunoaffinity binding uses different antibodies and reagents, there is also a chance of cross-contamination into the isolated exosomes, which would create inviable samples, therefore leading to variable loading rates [112, 113].

#### Polyethylene polymer precipitation

The precipitation method utilises the sample solubility factors to ultimately precipitate exosomes from the solution [114]. This method is increasing more common nowadays as precipitation reagents are relatively inexpensive and the method is easy to perform, hence providing an easy method of isolation without the need for specialised equipment [115, 116]. This method allows flexibility in the sample volume being used and commonly results in high exosomal yields, although variable purity because contamination may be an issue, depending on the reagents being used [115, 116]. Further, there is a higher chance of particle aggregation in conjunction with this method compared to other more conventional methods such as ultracentrifugation or SEC and can be time-consuming while the sample precipitates [114–116].

#### Microfluidics

Exosome isolation using microfluidics is based upon microfluidic technology capable of particle detection, analysation, and isolation from various fluids [117]. In literature, it has been more commonly used for bodily fluids such as blood, saliva, and urine [117]. These techniques allow for particle separation through various means such as fluid acoustic models, lateral displacement of particles, and automated biocompatible chip technology which acts to separate cells based on their biological characteristics [117, 118]. These techniques can result in high exosome yields and purity and are often highly sensitive during particle sorting [117–119]. However, such methods are not commonly established and require specific instruments, which results in a highly expensive and experimental methodology. Hence, while such technology is useful, it is not necessarily viable when compared to less complicated and inexpensive methods such as ultracentrifugation, differential centrifugation, and SEC [117–119].

#### Ultrafiltration

The ultrafiltration method uses centrifugal force together with a cellulose membrane to isolate exosomes above the filter level, while other cellular debris are forced through the membrane into the waste component of the sample [120]. Ultrafiltration can consistently separate particles based on their size and molecular weight, while being relatively simple, fast, and inexpensive to perform [120, 121]. Considering, ultrafiltration produces high exosomal purity although variable yields due to the stringent nature of the required filter [120, 121]. While this method may be advantageous to perform, it becomes important to consider factors such as filter clogging and the potential loss of exosomes which are smaller or larger than the average exosome, leading to less overall exosomal yields [120, 121].

### Exosome loading methods

#### Incubation

The incubation method can be the most straightforward and simplest method to use for exosome cargo loading [122]. This is done by allowing the desired cargo to diffuse across a concentration gradient at a certain temperature (usually 37 °C) over a set time [122]. Since exosomes and the plasma membranes are mostly hydrophobic and lipid-enriched, many cargos (more so hydrophobic ones) can interact with the exosomal membrane and be engulfed into the exosome in a spontaneous manner [122]. Furthermore, exosomes contain a hydrophilic core, which can facilitate hydrophilic cargo loading, allowing for flexibility in cargo choice [123]. Incubation may be useful depending on the type of therapeutic cargo as it is usually less damaging to both the cargo and the exosomes, compared to other physical methods [122]. A previous study loaded curcumin into exosomes isolated from HEK293 cells for testing anti-inflammatory effects, in which loading efficiency was described to be 65.8%, denoting moderate encapsulation [122]. Paclitaxel (PTX) has also been loaded into exosomes via incubation and was found to be useful for maintaining the exosomal membrane to a higher extent compared to other methods, although did not harbour the same high loading efficiency as sonication [124].

Another study incubated bovine milk–derived exosomes with siRNA and found that while there was more consistency in maintaining the exosomal membrane, the loading efficiency was less than electroporation [124]. Overall, this method has previously been a popular choice, but newer methods tend to be preferred due to the relatively low cargo loading efficiency of incubation [101].

#### Electroporation

Electroporation is a physical method which loads cargo into exosomes by creating an electrical field that produces micro-pores on the exosomal membrane and increases permeability [101]. The electrical field is produced by exposing the exosome-cargo mixture to a series of electric pulses over a range of different voltages, using a set amount of capacitance [125]. Electroporation has commonly been utilised to load cargo such as nucleic acids, nanomaterials, and certain drug molecules [101]. However, it becomes important to consider the physicochemical properties of the medium and the cargo being loaded into the exosomes as exposure to electrical currents can often disrupt membrane integrity and the chemical composition of different compounds [125]. Electroporation has been often used for loading drug molecules as it provides superior loading efficiency compared to incubation and other related methods [126]. In example, the anti-cancer drug doxorubicin (DOX) has been loaded into exosomes isolated from MDA-MB-231 and HCT-116 cell lines using electroporation [127]. It was found that DOX had increased effects at the target sites of action and less overall toxicity compared to the medication by itself [127]. Although electroporation remains an effective method in relation to loading efficiency, it causes particle aggregation which can render exosomes and related cargo unusable to varying extents [128].

#### Sonication

Sonication is another physical method for loading therapeutic cargo into exosomes. This method works by using sound energy to permeabilise the exosome wall by forming nanopores [129]. This leads to fast and efficient loading of drugs, proteins, and other nanoparticles [129]. Sonication can be a useful method for exosome cargo loading as it is generally less detrimental to the cargo or the exosomal wall integrity compared to electroporation, although particle aggregation remains an issue [101]. The cytotoxic medication gemcitabine (GEM) has been loaded into exosomes isolated from pancreatic cancer cell line (Panc-1) cells using sonication [129]. A high loading efficiency of 11.68 ± 3.68% was reported, compared to the incubation method leading to lower loading efficiencies at 2.79 ± 0.72% [129]. Another recent study loaded exosomes with PTX and obtained high loading efficiency compared to incubation, with minimal disruption to the structural integrity of the cargo or the exosomal wall compared to electroporation [124].

Additionally, a recent study focussed on loading exosomes with human chorionic gonadotropin (hCG) by using sonication [130]. As endometrial exosomes contain bioactive molecules which promote implantation, human hCG was loaded into exosomes with the aim of improving endometrial receptivity [130]. The study found that sonication was much more effective in loading efficiency compared to the freeze–thaw cycle method, with loading capacities of 710.05 ± 73.74 and 245.06 ± 95.66 IU/mg respectively [130]. Hence, sonication proves to be a solid method for exosome loading–however, particle aggregation and exosome membrane damage can be problematic due to the intensities of prolonged sound energy output. These factors may be somewhat mitigated if experimental parameters were optimised for the desired cargo, relating to cargo-specific such as size and zeta-potential [101]. Further research would be required to ascertain the optimal sonication settings in conjunction with loading different therapeutic cargo which may have distinct variabilities, such as between drug molecules and protein particles.

#### Transfection

The transfection method utilises certain reagents for the induction of specific plasmids into cells to ectopically express targeted proteins, peptides, or nucleic acids which may be loaded into exosomes afterwards [131]. In example, this method has been popular for efficiently and safely loading miRNA into exosomes for therapeutic benefits, such as miR*-*122 by transfecting mesenchymal stromal/stem cells (MSCs) with miR*-*122-expressing plasmids [131]. This resulted in exosomes highly expressing miR*-*122 and having therapeutic potential in treating hepatocellular carcinoma [131]. Further, siRNA has also been loaded through using human embryonic kidney 293 (HEK293) cells for treating chronic myeloid leukaemia, which becomes important as gene-silencing therapy, rather than drug therapy [132].

Additionally, transfection has been used with HEK293 cells to create exosomes loaded with catalase mRNA for targeting CNS cells in treating Parkinson’s disease (PD) [133]. Neurological diseases become a highly relevant therapeutic target for exosomes as they can pass the BBB effectively and deliver therapeutic cargo more safely and with high bioavailability [133]. Another recent study transfected bovine milk–derived exosomes with hsa-miR-148a-3p with an aim to examine the therapeutic benefit in relation to miRNA-based therapy [10]. The study found that cells treated with has-miR-148a-3p underwent changes in processes relating to insulin response, protein kinase B signalling, and cholesterol homeostasis, among various other alterations [10]. It was also shown that exosomes can efficiently be used for miRNA therapy with minimal off-target effects through a high absorption of exosomal cargo in HepG2 and Caco-2 cell lines [10]. Overall, transfection seems to be an efficient method for the safe and easy loading of cargo into exosomes, although issues can arise relating to high production costs, chemical impurities, and reduced loading efficiency [134]. Further, since it is difficult to completely remove the transfecting agent, there is a risk of haemolytic toxicity to cells if the transfection reagent is not properly removed [134].

#### Extrusion

This is another physical loading method which works by squeezing the exosome-cargo mixture within an extruder to induce membrane fusion–this must be done more than once, depending on the cargo, to effectively load exosomes [101]. Extrusion has been previously used to load exosomes isolated from a mouse macrophage cell line (Raw 264.7) with catalase for therapeutic use in varying PD models [8]. Additionally, another study loaded porphyrins into exosomes and compared extrusion to the electroporation and saponin-assisted loading methods [135]. It was found that extrusion was extremely efficient and comparable to sonication and saponin-assisted loading, although created a distinctive change in the zeta-potential of the loaded exosomes [135]. While extrusion has been found to generally produce high cargo loading efficiencies, the physical force used in this method may disrupt the exosomal surface membrane structure [135]. This may promote instability or alter certain intrinsic properties, such as the favourable immunogenicity profile of exosomes, which would make it visible to the immune system upon entry into the body [135]. Moreover, extrusion has been used to load clodronate disodium–loaded (CDL) liposomal vesicles into exosomes and thereby creating a hybrid, where the exosomes were previously isolated from the murine fibroblast cell line L-929 [136].

Although extrusion has been shown to provide relatively high loading rates, a prominent limitation of extrusion remains the potential of cytotoxicity due to the changes in the exosomal zeta potential which occurs upon loading exosomes with the desired cargo [135]. It was shown that the zeta potential was stable between that of the exosome and the CDL liposome, indicating that a hybrid structure may provide more stability within the exosome. However, it has been reported that significant changes to the exosomal zeta potential are more common with the extrusion method and causes the exosome itself to become cytotoxic to target cells, thereby nullifying potential therapeutic benefits of loaded cargo [135]. It is important to consider how this limitation may be mitigated if the desired therapeutic cargo and the exosome have similar zeta potential levels. Further research would be required to optimise ideal experimental conditions as to how many extrusion cycles may be needed and to avoid potential damage to the exosome and the cargo.

#### Freeze–thaw

The freeze–thaw reconstitution procedure is a rapid and well-established method which has been previously used for liposomal formation [101]. In example, the exosomes and the cargo of interest may be briefly frozen at −80 °C and then thawed to the desired temperature, which is usually around room temperature at ~22 °C. This method has shown higher loading efficiency in comparison to incubation by loading catalase into exosomes isolated from Raw 264.7 cells with multiple freeze–thaw cycles, with rates of 14.7 ± 1.1% regarding freeze–thaw and 4.9 ± 0.5% for incubation [8]. However, a recent study isolated exosomes from human-derived endometrial stem cells (hEnSCs) and found that utilising the freeze–thaw method for loading atorvastatin into exosomes obtained only a 10% loading efficiency [137]. This was lower than other methods tested such as incubation (25%), sonication (20%), and incubation with the permeabilisation reagent Tween-20 (28%) [137]. The study also stated that multiple freeze-thawing cycles were commenced using exosomes exposed to atorvastatin, and that the parameters were set to −80 to 37 °C [137]. It is possible that the variability in loading efficiency may be due to the parametric changes between experimental studies and that adjusting the freezing and thawing temperature may increase or decrease loading efficiencies. Freeze-thawing is cost-effective and relatively simple to preform; however, loading efficiencies have been found to be inconsistent and particle aggregation has been an outlying issue after repeating the cycles enough times to load the desired cargo [138]. Depending on the physicochemical properties of the relevant cargo, potential damage may be caused to the molecules due to the repeated cycles of freezing and thawing. Overall, such damage to exosomes may hinder therapeutic effects when certain molecules are exposes to rapid fluctuations in temperature.

#### Surfactant permeabilisation

Utilising a surfactant such as saponin or Tween-20 works through dissolving fatty membrane molecules (cholesterol) and thereby creating micropores on the exosomal membrane to help facilitate cargo loading, without destroying the phospholipid bilayer [139]. Surfactant-based loading has been found to be useful for resulting in high loading efficiencies compared to passive loading without the use of a surfactant [140]. This is because saponin assists with the overall loading of large and small molecules [140]. A recent study isolated exosomes from a mouse neural progenitor cell line (C17.2) and utilised Tween-20 to assist with the loading of a protein (mCherry) into exosomes for transfer through the BBB [140]. It was found that the protein loading was significantly increased in the Tween-20 group compared with the non-permeabilised exosomes [140].

Furthermore, saponin permeabilisation together with electroporation was used to load exosomes isolated from various cells (MCF7, Caco-2, PC3, and HepG2) with different phytochemicals (myricetin = 66.89 ± 8.29%; soyasaponin αg = 17.46 ± 3.83%, and soyasaponin βg = 19.69 ± 4.37%) [141]. It was discussed that the highest loading efficiencies were in the permeabilised groups compared to the electroporation only group [141]. However, it was noted that exosomes which are permeabilised with saponin were more likely to attract irreversible membrane damage following the application of voltage–indicating that while efficient in loading, the damage to the exosomal membrane may render the loaded exosomes unusable and therapeutically inactive. Although this method produces efficient cargo loading, the drawbacks include the permanent disruption of the exosomal membrane, which would cause intracellular instability. Further, permeabilisers such as Tween-20 and saponin can be cytotoxic at relatively minimal levels–meaning that using too much may cause unwanted effects within the body (refer to Table 5 for a summary) [138].

**Table 5:** The different methods of exosome loading, most appropriate cargos, advantages, and relevant limitations

**Method**	**Type of cargo**	**Advantages**	**Limitations**	**References**
**Incubation**	Drug molecules (PTX), proteins (size-limitations), siRNA, miRNA, shRNA, Curcumin	Simple method, cost-efficient, easy to perform	Low loading efficiency, exosomal wall disruption, some cargo (drug molecules) may be sensitive to temperature fluctuations	[83, 84, 101]
**Electroporation**	Proteins, drug molecules (PTX, DOX), GEM, siRNA, miRNA, macromolecules, synthetic molecules (metal mixtures)	Cost-efficient, good for loading larger molecules, high loading efficiency, easy to perform	Exosomal wall disruption, can largely affect the zeta-potential of cargo, particle aggregation, cannot load hydrophobic molecules efficiently	[86–88, 101]
**Sonication**	Proteins, drug molecules (PTX, DOX, GEM) catalase, siRNA, miRNA, DNA, ssDNA	High loading efficiency loading molecules, can load larger molecules	Disruption of the exosomal wall, particle aggregation, sound waves may alter molecule composition, not very efficiency for hydrophobic molecules	[89–91, 101]
**Transfection**	siRNA, miRNA, DNA, endogenous molecules	Simple to perform, can produce very high loading rates	Cytotoxicity from transfection agent, may be difficult to wash transfection agent, mixed loading efficiencies, not suitable for larger molecules, expensive to perform	[74, 92–95, 101]
**Extrusion**	Drug molecules (PTX), miRNA, siRNA, curcumin, catalase	Simple to perform, cost-efficient, can produce high loading efficiencies	Not suitable for larger molecules, damage to the exosomal wall is often irreversible, may disrupt the cargo	[8, 96, 101]
**Freeze–thaw**	Drug molecules (PTX, DOX), catalase, miRNA, siRNA	Simple to perform, cost-efficient, can fuse membranes	May cause irreversible damage to the exosomal wall and cargo, not suitable for larger molecules, mixed loading efficiencies, particle aggregation rates are higher	[8, 98, 99, 101]
**Surfactant permeabilisation**	Drug molecules (PTX, DOX), catalase, siRNA, miRNA	High loading efficiency, cost-effective, easy to perform, materials are readily available	May be difficult to completely wash away surfactant, many surfactants can cause hemolysis and cytotoxic effects, can cause extensive damage to the exosomal structure, not all cargo molecules can mix with surfactants	[99–102]

## Exosomal therapeutics and targeted cargo delivery

Important aspects to consider when discussing exosome cargo loading are the therapeutic viability of loaded exosomes and the possibility of cargo targeted delivery to the intended site of action, thereby avoiding off-target effects and reducing overall side effects. A method to examine the viability of loaded exosomes may be to test the effect of which exosomes loaded with therapeutic cargo would have on cells versus only the cargo or exosomes [142]. One study utilised drug-resistant A2780/DDP cells to test the effectiveness of using cisplatin-loaded exosomes against the medication by itself to determine if therapeutic viability had been increased [142]. The study found that by using the cisplatin-loaded exosomes, the cytotoxicity of cisplatin was increased by a factor of 3.3 in the drug-resistant cells and a factor of 1.4 in the drug-sensitive cells versus the medication by itself [142]. Another study tested therapeutic viability through loading blood-derived exosomes with dopamine for the treatment of PD [143]. It was found that there was a > 15-fold increase in the bioavailability of dopamine for distribution in the brain through utilising exosomes as delivery platforms [143]. As previous research has shown, delivering loaded exosomes can prove to be efficient in maintaining drug therapy, potentially avoiding factors such as first-pass metabolism, limited bioavailability, and moving through barriers such as the BBB [8, 142, 143].

Considering the clinical importance of exosome cargo loading, it becomes necessary to question the viability of targeted delivery to the intended site of action. Currently, some strategies for manufacturing targeted exosomes includes isolating unmodified exosomes from the specific tissue of interest and taking advantage of their intrinsic alignment to the organ or tissue in question [144]. Other methods may involve modifications of the exosomal surface such as the removal or addition of certain adhesion proteins such as integrins [145]. One study investigated the effect of overexpressing an exosomal membrane protein (Lamp2b) to increase the targeting rate towards integrin αvβ3-positive anaplastic thyroid carcinoma cells for enhanced doxorubicin delivery [145]. The study found that the overexpression of Lamp2b increased the cargo delivery to the target cells and resulted in a significant reduction in tumour size in 8505C xenograft mouse models, with minimal side effects [145]. Relatedly, another study explored targeted delivery for anti-inflammatory effects after cerebral ischemia [146]. The authors isolated exosomes from a human neural progenitor cell line (ReN cells) and attached specific targeting ligands to the exosomal surface by designing a recombinant fusion protein [146]. The result was the significant inhibition of site-specific inflammation in mouse models and was shown to induce minimal off-target effects [146]. Thus, while exosomal loading is important, targeted delivery is also crucial for optimising therapeutic outcomes by reducing off-target side effects and increasing bioavailability at the intended site of action.

## Remaining limitations

Considering the promising applicability of exosomes in diagnosing and treating reproductive disorders, various challenges remain for the translation from the laboratory to the clinic. Firstly, there are limitation surrounding vesicle isolation and further determining whether the isolated exosomes would be viable for clinical use. This can be due to the inconsistencies related to the isolated particle number, relevant morphology, and the source of isolated exosomes. In example, exosome characterisation may need to be completed using exosomes from both non-malignant and malignant cell lines for acceptable comparison if utilising exosomes for cancer therapy related research [147]. Further, techniques such as electron microscopy and western blotting may be useful for confirmation of presence and composition, although they provide no quantitative information [147]. Relatedly, nanoparticle tracking analysis is useful for estimating the number of exosomes present in a sample, although it does not provide information regarding the cell of origin and may also be hindered by limitations in measuring Brownian motion over a set period [148]. Another prominent challenge is the issue of particle aggregation following certain loading methods such as electroporation and sonication. This may result in less efficient cargo delivery if particles are aggregated together, and it may render some compounds inactive, therefore making them inappropriate for therapeutic delivery.

Despite the issue of particle aggregation, both electroporation and sonication have been shown in previous research as highly efficient loading methods [94, 97, 102, 122]. Due to the rapid changes on the exosomal surface while exosomes are near each other, particles can align together and fuse during the reformation of the exosomal wall. Various efforts to mitigate particle aggregation have not been explicitly addressed in recent literature. However, it has been noted that various incubation protocols following loading could separate membranes alongside utilising less intense voltages and sound waves, depending on the desired cargo [77].

Another prominent limitation is the variability in exosomal yield and purity related to isolation techniques. While some isolation techniques provide higher yield and purity than others, inconsistencies in methodologies, equipment, and human error prove it difficult to quantify which method is most appropriate to use [149, 150]. For example, the ultracentrifugation method is efficient for exosome isolation although often produces mixed results with protein contaminants and modifications to exosome structural integrity [149, 150]. Method optimisations would be required to infer the optimal conditions for different types of isolations [149, 150]. Alongside this is the inconsistency in cargo loading efficiencies of various methods such as electroporation, sonication, and extrusion, including the methods for measuring cargo loading efficiency. The differences in parameter outputs (electroporation, sonication) and the possible differences in equipment and technique (extrusion), along with inconsistencies in determining loading efficiencies, may be ongoing issues for the reproducibility of reliable results in future literature. However, the type of cargo also plays a role in determining loading efficiencies. For example, the variance in zeta-potential of the cargo and the exosome in response to loading treatment may hinder encapsulation, such as when attempting to load certain drug molecules (PTX) [36]. Moreover, the zeta-potential can be a determinant of the colloidal stability of exosomes and their actions at target cells, meaning that if significantly altered, detrimental effects may be experienced at target sites [36]. Relatedly, it has been reported that a positive surface charge (zeta-potential) can change the immunogenicity profile of exosomes, while a negative surface charge increases compatibility and the efficient delivery into target cells [130]. To address this potential issue, it may become beneficial to tailor parameters and methodologies relative to the specific physicochemical properties of the desired cargo, such including the zeta-potential and other factors relating to size, lipophilicity, and compatibility. Considering, testing various parameters against loading efficiencies has not been actively included in recent literature–which highlights the importance of method optimisations in response to experimental reproducibility.

## Conclusions and finalising thoughts

It is evident that exosomes are important in various physiological states, including during the reproductive cycle. The potential of exosomal research pertaining to human reproduction is extensive, although still faces many different questions in which future research must clarify. Such includes the challenge of utilising exosomes as therapeutic tools in many of the complications during pregnancy, both as diagnostic tools and as therapeutic treatment options. The question also remains as to how exosomes can be efficiently loaded and made clinically viable on a consistent basis, thereby allowing mass production for use in various clinical settings. The notion of isolating exosomes, therapeutic cargo loading, and further using exosomal biomarkers as early diagnostic and clinical tools for the detection and possible treatment of reproductive diseases is both exciting and perplexing. However, it is still unknown as to (1) how exosomes may be utilised effectively as diagnostic tools, both from a time-critical, ease-of-access, and financial point of view; (2) how loading exosomes with specific cargo can be clinically viable in terms of using exosomal markers to target sites of disease action; and (3) whether exosomal contents (such as miRNA, proteins, etc.) are different in the various stages of reproduction and if any differences could affect exosomal markers when considering targeted delivery.

Furthermore, an enduring challenge in exosome research remains the unanswered questions relating to the reproducibility of research and the optimisation of loading methods to ensure higher loading efficiencies. Following these challenges, the potential towards a solid groundwork for establishing exosomes as fast and efficient diagnostic tools and therapeutic delivery vehicles in pregnancy disorders and other clinical settings may be in reach. In turn, optimising reproducible methods in future research may help in paving the pathway towards the eventual use of exosomes in multiple areas of clinical research and practice. Thus, it becomes important to examine not only exosomal cargo loading, but also the various mechanisms relating to exosomal isolation, cellular uptake, and their function as biomarker carriers. While the field of reproductive extracellular vesicle research is abundant with potential, it is important to pursue the different hypotheses and extend the field further into real-world clinical applicability.

## Data Availability

Any data sets which were generated and/or analysed during this study are available from the corresponding author upon reasonable request.
